# ExTaxsI: an exploration tool of biodiversity molecular data

**DOI:** 10.1093/gigascience/giab092

**Published:** 2022-01-25

**Authors:** Giulia Agostinetto, Alberto Brusati, Anna Sandionigi, Adam Chahed, Elena Parladori, Bachir Balech, Antonia Bruno, Dario Pescini, Maurizio Casiraghi

**Affiliations:** University of Milano-Bicocca, Department of Biotechnology and Biosciences, Piazza della Scienza 2, 20126 Milan, Italy; Istituto Auxologico Italiano - IRCCS, Via Giuseppe Zucchi 18, 20095 Cusano Milanino, Italy; Università degli Studi di Pavia, Dipartimento di Scienze del Sistema Nervoso e del Comportamento, Via Agostino Bassi 21, 27100 Pavia, Italy; Quantia Consulting srl, Via F. Petrarca 20, 22066 Mariano Comense, Italy; University of Milano-Bicocca, Department of Biotechnology and Biosciences, Piazza della Scienza 2, 20126 Milan, Italy; University of Milano-Bicocca, Department of Biotechnology and Biosciences, Piazza della Scienza 2, 20126 Milan, Italy; Institute of Biomembranes, Bioenergetics and Molecular Biotechnologies (CNR), Via Amendola 122/O, 70126 Bari, Italy; University of Milano-Bicocca, Department of Biotechnology and Biosciences, Piazza della Scienza 2, 20126 Milan, Italy; University of Milano-Bicocca, Department of Statistics and Quantitative Methods, Piazza dell'Ateneo Nuovo 1, 20126 Milan, Italy; University of Milano-Bicocca, Department of Biotechnology and Biosciences, Piazza della Scienza 2, 20126 Milan, Italy

**Keywords:** biodiversity, data visualization, molecular data, database, data integration, taxonomy gaps

## Abstract

**Background:**

The increasing availability of multi-omics data is leading to regularly revised estimates of existing biodiversity data. In particular, the molecular data enable novel species to be characterized and the information linked to those already observed to be increased with new genomics data. For this reason, the management and visualization of existing molecular data, and their related metadata, through the implementation of easy-to-use IT tools have become a key point to design future research. The more users are able to access biodiversity-related information, the greater the ability of the scientific community to expand its knowledge in this area.

**Results:**

In this article we focus on the development of ExTaxsI (Exploring Taxonomy Information), an IT tool that can retrieve biodiversity data stored in NCBI databases and provide a simple and explorable visualization. We use 3 case studies to show how an efficient organization of the available data can lead to obtaining new information that is fundamental as a starting point for new research. Using this approach highlights the limits in the distribution of data availability, a key factor to consider in the experimental design phase of broad-spectrum studies such as metagenomics.

**Conclusions:**

ExTaxsI can easily retrieve molecular data and its metadata with an explorable visualization, with the aim of helping researchers to improve experimental designs and highlight the main gaps in the coverage of available data.

## Introduction

In recent years, studies investigating biodiversity at large scale have started to create and incorporate molecular data in biological databases. In particular, the spread of metagenomics studies (e.g., DNA metabarcoding) has contributed to an exponential increase in genomics data availability. Thanks to this large amount of new information it is possible to expand our knowledge and enhance our scientific investigation capacity in many fields of research [1], ranging from macro-ecology and ecosystem monitoring to food safety control, forensics applications, and microbiome identification [1–3]. Different groups of researchers emphasized the wealth of information collected in biological and molecular databases, with the aim of improving data usefulness and reusability [4, 6, 7]. Therefore, building experimental designs that consider the totality of the data present in such databases would increase the efficiency of these studies and lead to more robust results [8,9].

Biodiversity data retrieval and exploration are listed among the challenges of “big data” science, forcing researchers to use information technology (IT) tools for their management. In particular, the interpretation of results derived from metagenomic experiments, requiring computational pipelines and IT infrastructures that are improving over time, is strongly linked to the availability of pre-existing data stored in online databases (e.g., ENA [www.ebi.ac.uk/ena] and NCBI [https://www.ncbi.nlm.nih.gov/]).

In this context, data visualization represents an effective strategy not only to aggregate and expose the research results but also to guide advanced scientific investigations [10,11]). At this moment, reference databases, where molecular and taxonomic data are freely explorable and regularly updated, exist only for a few molecular markers, such as SILVA for 16S and 18S genes [12], BOLD for animals and plants [13], or UNITE for the Fungi domain [14]. However, these data resources are not representative of all the genomic and taxonomic diversity collected to date. On the other hand, although GenBank still summarizes the majority of genetic data and their related metadata currently available [15–17], such information is not always easy to access without specific bioinformatics and IT skills, which constitute a limiting factor to a large audience of scientists.

With the aim to help biologists to improve their experimental designs and to promote data exploration and exploitation, we have developed a tool, ExTaxsI (Exploring Taxonomy Information), that can facilitate molecular data integration with its associated taxonomy and metadata, eventually retrieved from heterogeneous sources. Moreover, its easy-to-use interface would greatly help researchers and practitioners in the visualization of either query results obtained from the NCBI Nucleotide database (molecular sequences and their metadata) or external user-defined data based on standard taxonomy notation.

To our knowledge, tools that provide user-friendly instruments to download and explore taxonomic data from NCBI have not been completely implemented yet. Currently, there are only a few tools that perform part of this task, focusing on slightly different goals. For example, NCBImeta [18] allows NCBI databases to be queried via command line scripts, favoring in particular the exploration of metadata associated with the records, but it does not integrate scripts or libraries to promote data visualization and exploration, neither does it incorporate the NCBI taxonomy reference database [19]. On the other hand, TaxonTableTools [20] includes workflows to analyse data produced by the user, focusing on DNA metabarcoding common approaches. ExTaxsI, instead, implements NCBI data retrieval to create formatted databases useful for taxonomy assignment methods and explore the results from a taxonomic and molecular point of view. In particular, it is linked to the NCBI taxonomy database [19] and ETE toolkit [21], in order to produce standard formats readable by most common software packages that deal with taxonomic information [22–27], such as the QIIME2 platform [22]. The tool is applicable to any molecular marker, gene name, or taxonomic group data, where it is also possible to create a non-standard marker genes database usable in metagenomic/metabarcoding taxonomic assignment tools [22]. In addition, thanks to the integration of the NCBI query tool [28], ExTaxsI can reorganize personal datasets in a standardized format to easily describe taxonomic variability and geographic provenance of records.

## ExTaxsI at Work

**Figure 1:**
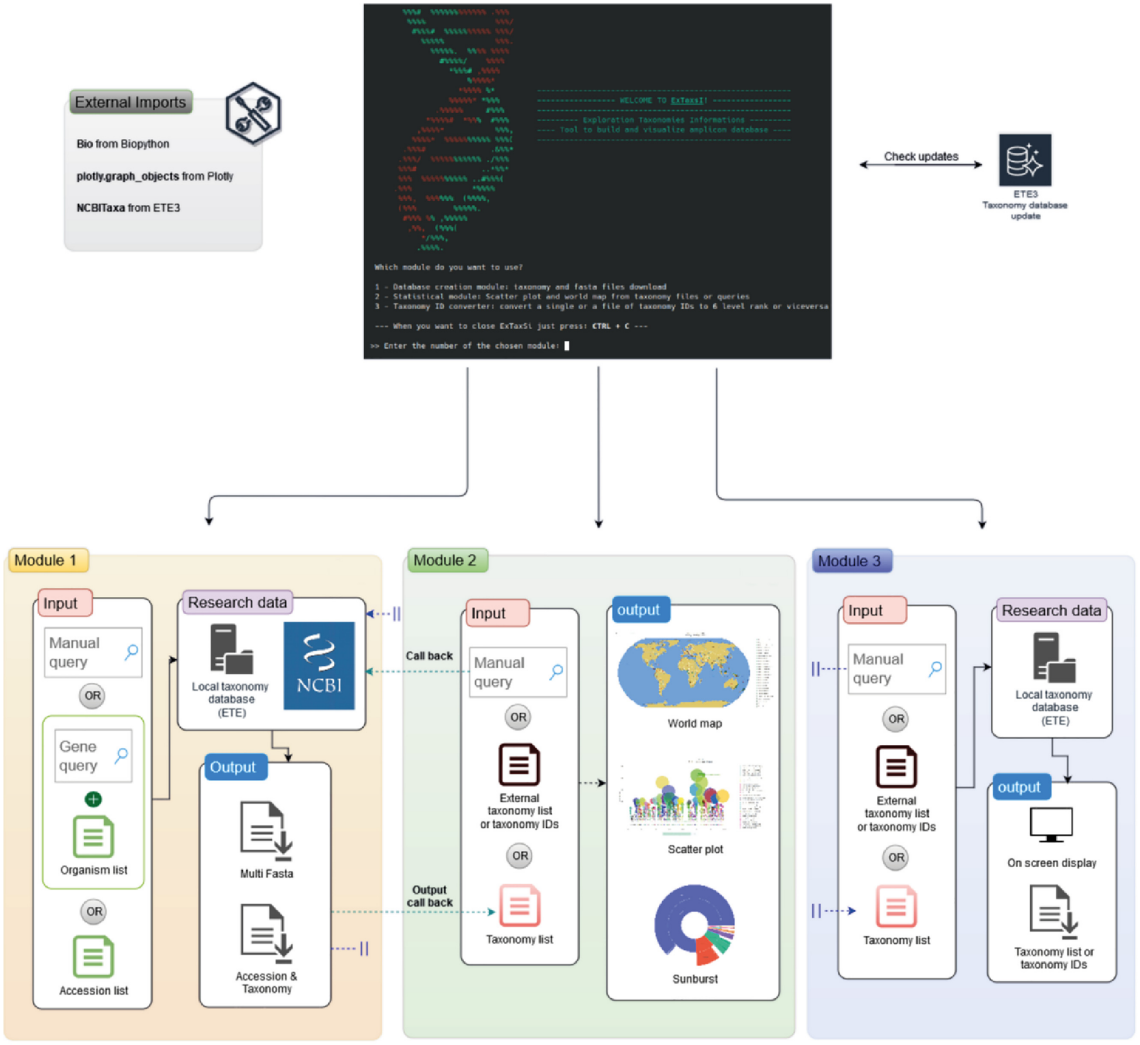
: ExTaxsI pipeline: module 1 (orange) searches and creates files and databases; module 2 (green) processes georeferenced or taxonomic data for the creation of graphs and plots; module 3 (blue) converts taxonomic names into NCBI taxonomy ID (txid) and vice versa.

ExTaxsI is a bioinformatic open-source tool aimed to elaborate and visualize molecular and taxonomic information via a simple interface. It is developed in Python 3.7 both as command line and as a Python library. The command line scripts are available through a user-friendly console, as they are built to make the tool interactive, helping the users via questions and explanations. In contrast, the Python module was built for advanced users to facilitate its integration into specific analytical pipelines (e.g., genomics, metagenomics). As illustrated in Fig. 1 this open-source instrument, starting from a list of taxa or gene name/s, allows the user to (i) search for taxonomic, genetic, and biogeographical data through NCBI databases, (ii) create a local and formatted nucleotide sequence (FASTA format) dataset, as well as (iii) their related taxonomy classification paths/datasets, thanks to the integration of NCBI taxonomy data, (iv) generate lists of genetic markers coming from different studies, and finally (v) produce interactive plots starting from NCBI query search results or directly from offline taxonomic files, including representative graphs for the exploration of taxonomy and refinement of biogeographical data by creating geographical maps with the locations of the species analyzed (Fig. 1). It is important to note that ExTaxsI outputs are compatible with other tools for taxonomic assignment purposes [23–27], such as the QIIME2 platform [22].

The communication with the NCBI server is mediated by the Entrez module [28], implemented in the Biopython library [29], which allows query results to be searched, downloaded, and parsed. To help NCBI interaction, for requests <2,500, the search key consists of a single query; otherwise the query is split into groups of 2,500, generating temporary files that are then merged into a single output file at the end of the process.

The ETE toolkit was used to handle taxonomy [21]. In particular, ETE allows a local taxonomy database to be created and kept up to date by extrapolating the 6 main ranks (phylum, class, order, family, genus, and species). If the organism is poorly described or is an unknown species, the NCBI taxonomy ID (txid) of its ancestor (known as parent txid) in the ETE taxonomic tree is then used and converted into its corresponding scientific name. It is important to underline that all queries are carried out locally, avoiding unnecessary online response delays. Finally, the extracted data are visualized through scatter plot and interactive sunburst chart to explore taxonomy and through world map plot to plot geographic metadata.

### Use cases

Because ExTaxsI is a taxonomy-focused data exploration tool, we designed 3 possible scenarios of variable complexity to challenge it with increasing taxonomic variability and dimension of accession entries. The first scenario hypothesizes a query to explore data with low taxonomic variability and a high number of expected entries (1 species, >300,000 entries). The second scenario provides high taxonomic variability and a large expected number of entries (∼500 species, >300,000 entries). The third and more complex scenario explores a complete case study with taxonomic input intersected by molecular data. Considering the case studies of the first 2 scenarios, we focused on taxa of interest in marine fisheries: (i) the codfish species (*Gadus morhua*), which is of global economic importance, and (ii) its taxonomic group at the order level, Gadiformes, which supports long-standing commercial fisheries and aquaculture. These 2 case studies evaluate the capacity to explore data and to fill in the geographic distribution of species, prospecting also the available gene information to perform a genetic survey (e.g., in a potential DNA metabarcoding study).

With the third use case, we aimed at demonstrating the flexibility of ExTaxsI in different contexts: a genetic exploration of the available data in NCBI associated with the SARS-CoV-2 virus—a very recent topic that involved many research groups, leading to huge amounts of data collected and deposited in public repositories [30]. A large-scale exploration of data related to this topic could improve the reliability of the results and provide valuable evidence to inform public health decision making, both now and in the future.

#### Insights into 2 taxonomic groups of commercial interest

The first scenario is the case of *Gadus morhua* (family: Gadidae; order: Gadiformes), the Atlantic cod. *G. morhua* is a large, cold-adapted teleost fish that supports long-standing commercial fisheries and aquaculture [31–35].

**Figure 2:**
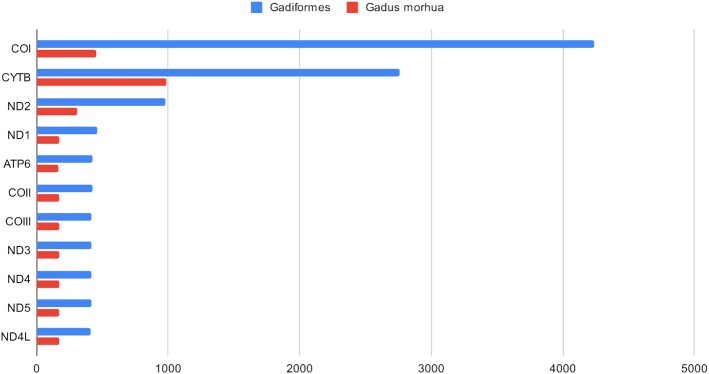
: Gene distribution of accessions with available “gene" tag information among *Gadus morhua* and Gadiformes taxons.

**Figure 3:**
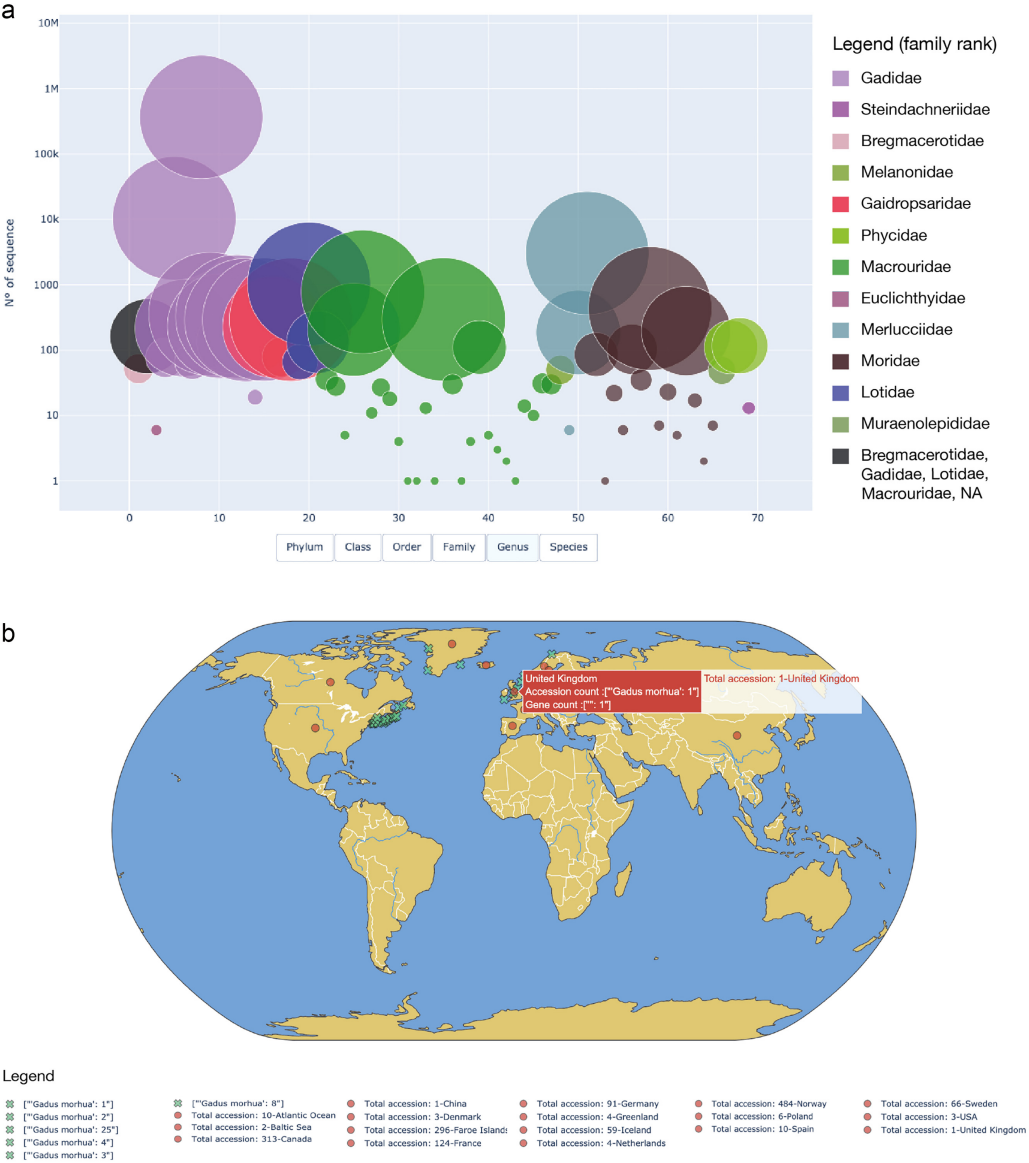
: (a) Scatter plot of Gadiformes accessions representing sequence abundances among families; (b) world map plot of *Gadus morhua* distribution considering geographic metadata extracted from the records.

ExTaxsI retrieved a total of 367,455 accessions (18 June 2021) using the Taxonomy ID through the following query: “txid8049[ORGN]” (where 8049 is the *G. morhua* NCBI txid). Only 54,061 entries showed a “gene" tag that could be investigated by ExTaxsI. As it is a unique species, we decided to represent the results obtained from a gene survey (Fig. 2) and the world map plot (Fig. 3).

Regarding gene distribution, the most abundant gene is *CYTB* (cytochrome b, with 985 accessions), followed by *COI* (cytochrome c oxidase subunit I; 455) and ND2 (311). These results are in line with those obtained by Knudsen and colleagues [32], where they personally developed specific primers for *CYTB* amplification because it is a widely used marker in fish molecular characterization. The remaining most abundant genes are the other *ND* portions and cytochrome oxidase fragments (*COIII* and *COII*), belonging to the mitochondrial genome. These results show the pronounced effort involved in sequencing “standard” DNA barcoding markers, while moderately sequencing larger portions of mitochondrial genomes. The remaining genes in the retrieved list and their relative accession frequency distribution (see the complete list in Additional File 1) demonstrate that many regions of the genome were investigated.

Geographically, the Gadidae family has a circumpolar distribution, comprising species occurring principally in northern and cool seas [31]. Furthermore, as reported by Jorde and colleagues, in Norway we can recognize 4 distinct stocks of the Atlantic cod: (i) the oceanic Northeast Arctic cod, (ii) coastal cod north of 62°N, (iii) coastal cod south of 62°N, and (4) a North Sea/Skagerrak stock, the most densely populated region in Norway [31]. This geographic distribution is partly visible via the metadata extracted by ExTaxsI, as shown in the world map plot in Fig. 3b (Additional File 2).

The second scenario takes as an example the Gadiformes Order (phylum: Chordata; class: Actinopterygii), a major group of organisms belonging to marine fisheries. It includes many important food fishes, variously marketed as cod, hake, grenadier, moras, moray cod, pelagic cod, codlet, and eucla cod [36]. A vast group, it comprises >500 species, which contribute to more than one-quarter of the world’s marine fish catch [36,37].

Via ExTaxsI, this order was explored using the query “txid8043[ORGN]”, yielding 389,640 accessions (where 8043 is the Gadiformes NCBI txid; 21 June 2021), where 61,249 showed the “gene" tag information. As a group spread on different taxonomic levels, both taxonomy and gene lists were created. In detail, to explore taxa distribution and accession abundances across the entire order, the tool created a scatter plot and sunburst plot in HTML format. Figure 3a shows genera across families via a scatter plot, while a sunburst plot and fully interactive plots showing the complete dataset are available in the Additional Files 3 and 4.

As shown in Fig. 3a, Gadidae is the most abundant family, represented by 381,460 accessions, followed by Merlucciidae (3,252) and Macrouridae (1,673). These results are in accordance with the literature because the Gadidae family is a primary marine, bottom-dwelling family of fishes in the Gadiformes order with great commercial importance [32,36].

Furthermore, considering the scatter plot in Additional File 3, the interactive visualization shows the taxonomy distribution among the available accessions, changing the rank dynamically as the user continues exploring. This feature revealed that the genus *Gadus* is the most abundant of the entire dataset, in which 94.3% of the accessions corresponded to *G. morhua*. This result is expected because *G. morhua* is documented to be a key species both in the North Atlantic ecosystem and commercial fisheries, with increasing aquaculture production in several countries [31].

Considering the genetic information obtained by ExTaxsI, a total of 28,850 unique genes were found from the 61,249 completely tagged accessions. A representation of the 10 most abundant genes is reported in Fig. 2, where at the first position the *COI* gene is placed, a widely used marker gene in DNA metabarcoding projects [32], dealing mainly with animal species identification [1], followed by *CYTB* and *ND2* [1].

Finally, these 2 case studies showed the ability of the tool to accurately portray the state of the art of the genetic information available in NCBI. Comparing the most abundant genes found among the records, it is possible to see a slight discrepancy between the 2 taxa explored (Fig. 2), highlighting the disclosures that the survey can report. In general, the detection of mitochondrial genes, coding for *COI* and *CYTB*, is in accordance with the reliability of these DNA barcodes, principally used in the discrimination of animal species [38–40]. To date, considering the subjects of our use cases, different studies have used *COI* or *CYTB* barcoding to identify seafood products and explore broad patterns in fish mislabelling [41–47].

In addition, these use cases higlighted the importance of extracting the geographical metadata from NCBI records. The completeness and the collection of such data can drastically improve biogeographic and ecological research, allowing not only exploration of sampling areas, but also improvement in phylogeography investigations, biodiversity monitoring, and environmental genomics strategies [1,48]. Moreover, the retrieved data showed an imbalance between the number of records and the number of explorable genes, which is in some cases due to the incompleteness of the “gene" tag. In recent years, genome sequences have started to play a key role in public repositories, making sequences available for sharing and reuse. The submission process can be challenging and errors can affect the availability and the quality of the data. For this reason, there is a wide interest in integrating standardized procedures into the annotation process [49] that can be enhanced by adopting FAIR principles and best practices to avoid error propagation in sequence databases [50,51], making the data fully explorable in the future.

#### Exploring biodiversity data in a pandemic outbreak: the case of SARS-CoV-2

The severe acute respiratory syndrome coronavirus 2 (SARS-CoV-2) is an enveloped, positive-sense, single-stranded RNA virus that causes coronavirus disease 2019 (COVID-19). RNA and structural proteins are included into virus particles mediating host cell invasion. After cell infection, RNA encodes structural proteins that make up virus particles. Virus assembly, transcription, replication, and host control are mediated by nonstructural proteins [52]. The pandemic linked to SARS-CoV-2 highlighted hidden virus reservoirs in wild animals and their potential to occasionally spill over into human populations [52]. A detailed understanding of this process is crucial to prevent future spillover events. As reported in the seminal article by Andersen and colleagues [53], the risk of future re-emergence events increases if SARS-CoV-2 pre-adapted in another animal species. SARS-CoV-2 probably originated from *Rhinolophus affinis* bats, with pangolin (*Manis javanica*) as intermediate host [53]. Recently, other animal species were posited to be possible intermediate hosts between bats and humans (54; 55). To date, ACE2 (angiotensin-converting enzyme 2), the receptor that binds to the receptor-binding domain of SARS-CoV-2 S protein [56], is reported as crucial in host invasion.

**Figure 4:**
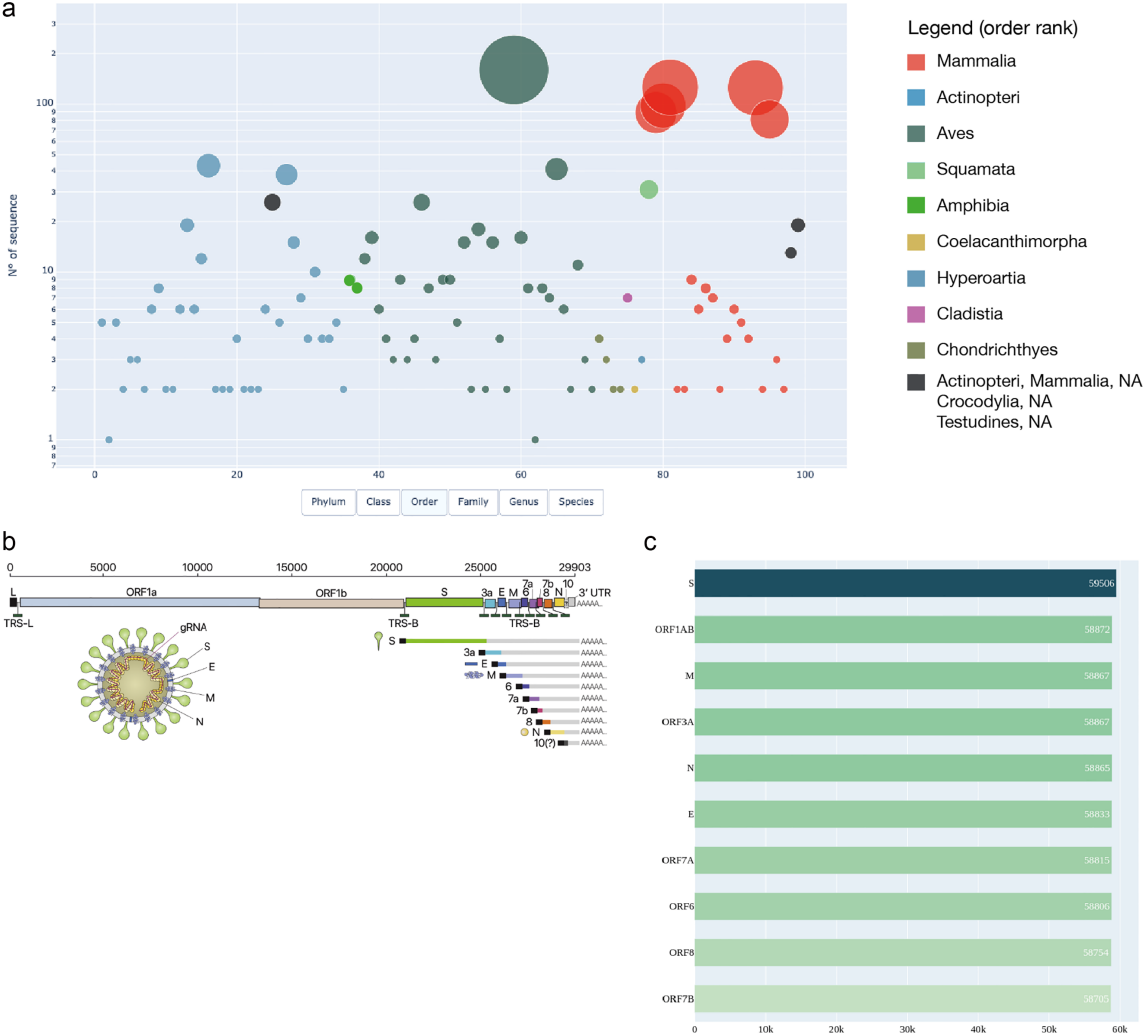
: (a) Scatter plot of ACE2 accessions representing sequence abundances among taxa at order level; (b) SARS-CoV-2 representation, from [59]; (c) gene distribution across accessions of SARS-CoV-2 data. gRNA: guide RNA; ORF: open reading frame; UTR: untranslated region.

To test our approach and explore the genetic information available in NCBI, we decided to extrapolate information about the *ACE2* gene from the Vertebrata taxonomic group, with the query “txid7742[ORGN] AND ACE2[gene]” (where 7742 is the specific Vertebrata NCBI txid). The results show that the *ACE2* gene is widely distributed throughout Vertebrata, as we obtained a total of 1,391 accessions (20 June 2021), distributed mainly among the Mammalian Class, with a high representation in Actinopteri and Aves groups (Fig. 4a; Additional Files 5 and 6 for an interactive exploration). In detail, Chiroptera, Primates, and Rodentia orders are the most represented, with 126, 125, and 81 accessions, respectively. In support of this molecular data survey, Luan and colleagues [57] analyzed the affinity of the 20 key amino acid residues in *ACE2* to S protein from mammal, bird, turtle, and snake and suggested that Bovidae (class: Mammalia) and Cricetidae (order: Rodentia) families should be included in the screening of intermediate hosts for SARS-CoV-2. In addition, thanks to the analysis of spike glycoprotein sequences from different animals, Dabravolski and Kavalionak [58] suggested that the human SARS-CoV-2 could also come from yak (family: Bovidae) as an intermediate host. In this context, ExTaxsI has the advantage of providing the complete list of taxa, allowing an exhaustive exploratory research by downloading all the sequences available for the query input, generating in turn the input for downstream analyses, such as the calculation of sequence similarities among different taxa. Furthermore, investigating shared features with other species can have important implications for understanding potential natural reservoirs, zoonotic transmission, and human-to-animal transmission. Noteworthy, the survey can give researchers an instrument to download specific data related to Covid-19, with a user-friendly approach, to explore the data interactively, including biodiversity-related information, and to design informed scientific experiments.

Last, we explored the data available for SARS-CoV-2 (Fig. 4) using the query “txid2697049” (where 2697049 is the specific Severe Acute Respiratory Syndrome Coronavirus 2 NCBI txid). Figure 4c shows the top 10 most abundant genes found in the retrieved entries and corresponding to a total of 773,293 accessions (28 June 2021). In particular, the most represented genes are *S* (59,506), the spike or surface glycoprotein fragment, *ORF1AB* (58,872), followed by *M* (58,867), *ORF3A* (58,867), and *N* fragments (58,865), the nucleocapsid protein. These results are in line with the recently published scientific data highlighting the functional aspects of viral proteins. Considering *ORF1AB*, several studies demonstrated its pivotal role among coronaviruses [60], providing a clinical target to break down SARS-CoV-2 infection [61]. In addition, the nucleocapsid phosphoprotein is involved in packaging the RNA into virus particles and protects the viral genome. For these reasons, it has been widely studied and suggested as an antiviral drug target [62,63]. The spike glycoprotein, in contrast, is located outside the virus particle, mediating its attachment and promoting the entry into the host cell. It also gives viruses their crown-like appearance. In the latest research, the S protein was found as an important target for diagnostic antigen-based tests, antibody therapies, and vaccine development [64, 65]. The entry of SARS-CoV-2 into host cells is mediated by further processes, e.g., the activity of the protease TMPRSS2 [66]. Also in this case, the use of ExTaxsI can unearth similar proteases in possible intermediate hosts, revealing new insights into the mechanism of infection.

As also documented by Khailany et al. [61], the emergent and huge amounts of data collected during the present pandemic necessitate a large-scale exploration. The rapid increment of data releases may give some important insights about SARS-CoV-2 behaviour in its host species, helping to improve not only our knowledge but also the design of appropriate prediction models of COVID-19 outbreaks and new target drugs.

## Conclusions and Future Directions

ExTaxsI provides an easy-to-use standalone tool able to interact with NCBI databases and personal datasets, offering instruments to standardize taxonomy information and visualize vast amount of data distributed on different taxonomic levels. It also provides interactive visualization plots, easily shareable through HTML formats.

The user-oriented interrogation of NCBI databases may help researchers involved in environmental genomics fields, from phylogeographic studies to DNA metabarcoding surveys, and also in projects related to human health, as demonstrated with the SARS-CoV-2 case study.

With this work, we hope to meet the needs of a broad group of researchers, providing an instrument easy to install either on common laptops or on high-performance servers and directly connected with NCBI databases. In parallel to the command line tool, a Python library containing all ExTaxsI functions has been implemented, favoring a direct incorporation of such functions into data analysis and exploration pipelines.

In addition, as data volume is increasing over time and NCBI databases still have a few constraints regarding the query results dimension and their retrieval time required, an automatic management of large queries will be implemented in future releases. Finally, we will also consider further data visualization strategies and additional metadata (e.g., GBIF country information) to enhance data interpretation and to provide comprehensive sets of relevant scientific-focused information. In our opinion, ExTaxsI's data management ability with its visual interactive exploration can really improve the experimental design phase and the awareness of the information available, facilitating data examination and sharing.

## Implementation

ExTaxsI is a bioinformatic tool aimed to explore, elaborate, and visualize molecular and taxonomic information via a simple user interface without specific bioinformatic or programming skills. The tool can be run, via command line interface, where the user is guided by the appropriate documentation of each script, avoiding the implementation of ad hoc Python code. ExTaxsI is developed in 3 separate modules, which can be used either interconnected as workflow or independently according to the user needs. The main modules are listed as follows: (1) Database creation, (2) Visualization, and (3) Taxonomy ID converter.

ExTaxsI is also available as a Python library that can be installed through pip (package installer for Python), containing the same functions and parameters as those of the command line tool. A detailed description of each module is provided below.

### 1. Database creation module

The module “Database" allows the user to create multi FASTA files composed of nucleotide sequences, taxonomic lists, gene names, and their related accessions, starting from either a single or a batch query mode using CSV/TSV input files (Fig. 1). After indicating the input type, it is possible to integrate the query with 1 or more gene names (or other details). This step allows the search to be restricted to NCBI databases if needed. In general, the output formats are (i) a multi-FASTA file (widely used format for molecular sequences) and (ii) text file in TSV format, with 2 columns composed by the accessions code followed by the taxonomy path of each accession at the 6 main levels separated by semicolons: phylum, class, order, family, genus, and species. When requested by the user, the output file of genes names is provided in TSV format consisting of a table with 2 columns, the first a the list of genes, and the second, the frequency values of the respective genes found in the retrieved records. The tool also provides a summary table containing the most popular genes from a list of NCBI txids, accessions, or organisms. In addition, it is possible to create a bar plot with the top 10 of the summary table, downloadable as a PNG file.

### 2. Visualization module

The module “Visualization" allows the user to create interactive plots, starting from the “Database" module output or from external sources such as local files (e.g., Additional Files 3–5) containing taxonomic lists. Before producing the plots, a dialogue box will ask the user to choose a filter value on the data based on the frequency. If the chosen filter value is 0, the tool processes all the data. Otherwise, all the taxonomic units that have not reached the minimum value are inserted into an additional text file, specifically created with a name containing the filter used.

The available plots generated by ExTaxsI are (i) scatter plot (Additional File 3), (ii) sunburst plot (Additional File 4), and (iii) world map plot (Additional File 2). All figures created by the Visualization module can be downloaded as HTML format files. In detail, scatter plot uses taxonomy as input to produce a graph that indicates the quantity of each individual taxonomic unit; the interactive plot enables the user to (i) choose the taxonomic level to be displayed using the buttons located under the graph and (ii) hover over points to show details, such as the number of records within taxa, names of selected taxa, and name of the parent taxon. The plot also allows the user to compare more data on mouse-over, highlight an area of interest with the zoom function, and view a specific group or remove specific taxa from the graph. Sunburst plot, in contrast, starting from a taxonomy input creates an expansion pie that allows taxonomy to be explored by clicking on the taxonomic group of interest and showing the underlying taxa within a new sunburst plot. Also in this case, hovering over points shows the number of records within taxa. Regarding the world map plot, the initial input is processed to obtain geographic data. The tool exploits the “Country" metadata stored in the NCBI records to produce a map indicating the position of each entry. In this step, on the basis of the type of geographic data obtained, ExTaxsI divides results into 2 different arrays: (i) a specific array of coordinates (if the coordinates are present in the record) or (ii) a specific array of country names (if the coordinates are absent). It is also possible to add data from external sources to the map. In each created map, the coordinates are indicated by green crosses, and countries, by red circles. Thinking of multiple taxa plotting, each symbol can have a legend that summarizes the data downloaded with the same country name or coordinate description. Furthermore, it is possible to see both genes and counts available among the represented accessions.

### 3. Taxonomy ID converter module

This module allows NCBI txid to be converted into the 6 main taxonomy ranks and vice versa (phylum, class, order, family, genus, and species); it can convert single manual inputs or multiple inputs from a TSV/CSV file containing a list of txids.

## Availability of Source Code and Requirements

### Command line tool

There are no specific system requirements for the installation of ExTaxsI; however, for the correct functioning of the software we suggest a minimum of 4 GB of RAM. To successfully run ExTaxsI, the following Python libraries must be installed: Biopython [29], NumPy [67], SciPy [68], Matplotlib [69], ipython [70], Pandas [71], SymPy (https://www.sympy.org/en/index.html), nose (https://nose.readthedocs.io/en/latest/), genutils (https://pypi.org/project/genutils/), requests [72], and Plotly (https://plotly.com/), in addition to Plotly-Orca and ETE toolkit [21]. To install all the dependency-compatible versions, we provide a requirement list at the GitHub page https://github.com/qLSLab/ExTaxsI, with a detailed guideline to directly setting a conda environment.

### Python library

The Python library Extaxsi is available both in the Github page https://github.com/qLSLab/ExTaxsI/tree/master/library and in PyPI repository: https://pypi.org/project/extaxsi/

- Project name: ExTaxsI
- Project home page: https://github.com/qLSLab/extaxsi; https://github.com/qLSLab/ExTaxsI/tree/master/library; https://pypi.org/project/extaxsi/
- Operating system(s): Platform independent
- Programming language: Python
- License: GNU GPL version 3
- bio.tools ID: extaxsi
- RRID:SCR_021846

## Data Availability

Snapshots of our code and other data further supporting this work are openly available in the GigaScience repository, GigaDB [73].

## Supplementary Material

giab092_GIGA-D-21-00226_Original_Submission

giab092_GIGA-D-21-00226_Revision_1

giab092_GIGA-D-21-00226_Revision_2

giab092_Response_to_Reviewer_Comments_Revision_1

giab092_Reviewer_1_Report_Original_Submission Iddo Friedberg -- 9/22/2021 Reviewed

giab092_Reviewer_2_Report_Original_Submission Luiz Gadelha -- 9/23/2021 Reviewed

giab092_Supplemental_Files

## Additional Files

**Additional File 1:** Gene list in TSV format obtained through ExTaxsI for the species *Gadus morhua*. Gene counts were extracted from 367,455 accessions (query: “txid8049[ORGN]”; 18 June 2021).

**Additional File 2:** World map plot in HTML format created via ExTaxsI extracting the values of “Country" tag contained in 367,4553 accessions of *Gadus morhua* (query: “txid8049[ORGN]”; 18 June 2021). Coordinates are indicated by green crosses, and states, by red circles.

**Additional File 3:** Scatter plot in HTML format created via ExTaxsI extracting the taxonomy of 389,640 accessions of Gadiformes Order (txid8043[ORGN]”; 21 June 2021).

**Additional File 4:** Sunburst plot in HTML format created via ExTaxsI extracting the taxonomy of 388,603 accessions of Gadiformes order (txid8043[ORGN]”; 21 June 2021).

**Additional File 5:** Scatter plot in HTML format created via ExTaxsI extracting the taxonomy related to 1,391 accessions of ACE2 genes belonging to the Vertebrata taxonomic group (query: “txid7742[ORGN] AND ACE2[gene]”; 20 June 2021).

**Additional File 6:** Sunburst plot in HTML format created via ExTaxsI extracting the taxonomy related to 1,391 accessions of ACE2 genes belonging to the Vertebrata taxonomic group (query: “txid7742[ORGN] AND ACE2[gene]”; 20 June 2021).

## Abbreviations

ACE2: angiotensin-converting enzyme 2; BOLD: Barcode of Life Data System; COI: cytochrome oxidase I; COII: cytochrome oxidase II; COIII: cytochrome oxidase III; CSV: comma-separated values; CYTB: cytochrome B; ENA: European Nucleotide Archive; ETE: Environment for Tree Exploration; FASTA: text-based format for representing either nucleotide sequences or peptide sequences; HTML: Hyper-Text Markup Language; IT: information technology; NCBI: National Center for Biotechnology Information; ND2: NADH dehydrogenase 2; PNG: Portable Network Graphics; QIIME2: Quantitative Insights Into Microbial Ecology; RAM: random access memory; SILVA: High quality ribosomal RNA databases; txid: Taxonomy ID; TSV: tab-separated values; UNITE: Database and sequence management environment centered on the eukaryotic nuclear ribosomal ITS region.
