# Supplementary material for: ExTaxsI: an exploration tool of biodiversity molecular data
Source: Gigascience. 2022 Jan 25;11:giab092. doi: 10.1093/gigascience/giab092 (PMC8848311; doi:10.1093/gigascience/giab092)
Supplement: giab092_GIGA-D-21-00226_Revision_1 [file giab092_giga-d-21-00226_revision_1.pdf]

GigaScience

ExTaxsI: an exploration tool of biodiversity molecular data

--Manuscript Draft--

**Manuscript Number:**	GIGA-D-21-00226R1	
**Full Title:**	ExTaxsI: an exploration tool of biodiversity molecular data	
**Article Type:**	Technical Note	
**Funding Information:**	ministero dell'istruzione, dell'università e della ricerca (CUP: H42F16002450001)	Mr Maurizio Casiraghi
**Abstract:**	The increasing availability of multi omics data is leading to continually revise estimates of existing biodiversity data. In particular, the molecular data enable to characterize novel species yet unknown and to increase the information linked to those already observed with new genomic data. For this reason, the management and visualization of existing molecular data, and their related metadata, through the implementation of easy to use IT tools have become a key point to design future research. The more users are able to access biodiversity related information, the greater the ability of the scientific community to expand the knowledge in this area. Results: In our research we have focused on the development of ExTaxsI (Exploring Taxonomy Information), an IT tool able to retrieve biodiversity data stored in NCBI databases and provide a simple and explorable visualization. Through the three case studies presented here, we have shown how an efficient organization of the data already present can lead to obtaining new information that is fundamental as a starting point for new research. Our approach was also able to highlight the limits in the distribution of data availability, a key factor to consider in the experimental design phase of broad spectrum studies, such as metagenomics. Conclusions:ExTaxsI can easily retrieve molecular data and its metadata with an explorable visualization, with the aim to help researchers to improve experimental designs and highlight the main gaps in the coverage of available data.	
**Corresponding Author:**	Anna Sandionigi<br>Quantia Consulting srl<br>Milan, ITALY	
**Corresponding Author Secondary Information:**		
**Corresponding Author's Institution:**	Quantia Consulting srl	
**Corresponding Author's Secondary Institution:**		
**First Author:**	Giulia Agostinetto	
**First Author Secondary Information:**		
**Order of Authors:**	Giulia Agostinetto	
	Alberto Brusati	
	Anna Sandionigi	
	Adam Chahed	
	Elena Parladori	
	Bachir Balech	
	Antonia Bruno	
	Dario Pescini	
	Maurizio Casiraghi	
**Order of Authors Secondary Information:**		
**Response to Reviewers:**	Dear Mrs Nogoy,	

Powered by Editorial Manager® and ProduXion Manager® from Aries Systems Corporation

	Thank you for the opportunity to publish our work in the Giga Science Journal and thank you for your work during the review process.<br><br>As suggested by Reviewer #2 we have created a Python library which can be found here:<br><br>https://pypi.org/project/extaxsi/<br><br>A python notebook is provided in the Github web page (https://github.com/qLSLab/ExTaxsI/blob/master/library/functions_installation_and_tutorial.ipynb). All functions are described in the details in order to make the installation and the use clear.<br>The functions available in the library are the same as those present in the command line tool. However, we decided to maintain the command-line version because from the feedback collected from non-bioinformatic users it would seem that for a first approach the simple command line is more immediate.<br><br>Also, as suggested by Reviewer#1, we have revised the text, fixing the sentences with unclear syntax.<br><br>I conclude by thanking you for having contributed to the improvement of our work.<br>We remain at your disposal for any clarification<br><br>Kind regards<br><br>Anna
**Additional Information:**	
**Question**	**Response**
Are you submitting this manuscript to a special series or article collection?	No
**Experimental design and statistics**<br><br>Full details of the experimental design and statistical methods used should be given in the Methods section, as detailed in our Minimum Standards Reporting Checklist. Information essential to interpreting the data presented should be made available in the figure legends.<br><br>Have you included all the information requested in your manuscript?	Yes
**Resources**<br><br>A description of all resources used, including antibodies, cell lines, animals and software tools, with enough information to allow them to be uniquely identified, should be included in the Methods section. Authors are strongly encouraged to cite Research Resource	Yes

Identifiers (RRIDs) for antibodies, model organisms and tools, where possible.<br><br>Have you included the information requested as detailed in our Minimum Standards Reporting Checklist?	
**Availability of data and materials**<br><br>All datasets and code on which the conclusions of the paper rely must be either included in your submission or deposited in publicly available repositories (where available and ethically appropriate), referencing such data using a unique identifier in the references and in the “Availability of Data and Materials” section of your manuscript.<br><br>Have you have met the above requirement as detailed in our Minimum Standards Reporting Checklist?	Yes

LaTeX - Main Document (TeX file)
Click here to access/download;LaTeX - Main Document (TeX file);main.tex

OXFORD (GIGA)^n SCIENCE

GigaScience, 2017, 1–9

doi: xx.xxxx/xxxx
Manuscript in Preparation
Paper

PAPER

ExTaxsI: an exploration tool of biodiversity molecular data

Giulia Agostinetto[1,*], Alberto Brusati[2,*], Anna Sandionigi[3,†], Adam Chahed[1], Elena Parladori[1], Bachir Balech[4], Antonia Bruno[1], Dario Pescini[5] and Maurizio Casiraghi[1]

[1]University of Milano-Bicocca, Department of Biotechnology and Biosciences, Milan, Italy and [2]Istituto Auxologico Italiano, Milan, Italy and [3]Quantia Consulting srl, Milan, Italy and [4]Institute of Biomembranes, Bioenergetics and Molecular Biotechnologies (CNR), via Amendola 122/O, 70126, Bari, Italy and [5]University of Milano-Bicocca, Department of Statistics and Quantitative Methods, Milan, Italy

[*]These authors contributed equally to the work.
[†]corresponding author: anna.sandionigi@quantiaconsulting.com

Abstract

Background The increasing availability of multi-omics data is leading to regularly revise estimates of existing biodiversity data. In particular, the molecular data enable to characterize novel species yet unknown and to increase the information linked to those already observed with new genomics data. For this reason, the management and visualization of existing molecular data, and their related metadata, through the implementation of easy-to-use IT tools have become a key point to design future research. The more users are able to access biodiversity related information, the greater the ability of the scientific community to expand its knowledge in this area. **Results** In this paper we have focused on the development of ExTaxsI (Exploring Taxonomy Information), an IT tool able to retrieve biodiversity data stored in NCBI databases and provide a simple and explorable visualization. Through the three case studies presented here, we have shown how an efficient organization of the available data can lead to obtain new information that is fundamental as a starting point for a new research of interest. Using this approach, it was possible to highlight the limits in the distribution of data availability, a key factor to consider in the experimental design phase of broad spectrum studies such as metagenomics. **Conclusions** ExTaxsI can easily retrieve molecular data and its metadata with an explorable visualization, with the aim to help researchers to improve experimental designs and highlight the main gaps in the coverage of available data.

Key words: Biodiversity; Data visualization; Molecular data; Database; Data integration; Taxonomy gaps

Introduction

In recent years, studies investigating biodiversity at large scale have started to create and incorporate molecular data in biological databases. In particular, the spread of metagenomics studies (e.g. DNA metabarcoding) have contributed to an exponential increase in genomics data availability. Thanks to this large amount of new information it is possible to expand our knowledge and enhance our scientific investigation capacity in many fields of research [1], ranging from macro-ecology and ecosystem monitoring, to food safety control, forensics applications and microbiome identification [2, 1, 3]. Different groups of researchers emphasized the wealth of information collected in biological and molecular databases, with the aim to improve data usefulness and reusability [4, 5, 6]. Therefore, building experimental designs that consider the totality of the data present in

Compiled on: November 16, 2021.
Draft manuscript prepared by the author.

such databases could certainly increase the efficiency of these studies, and lead to more robust results [7, 8].

Biodiversity data retrieval and exploration are listed among the challenges of "big data" science, forcing researchers to use Information Technologies (IT) tools for their management. In particular, the interpretation of results derived from metagenomic experiments, requiring computational pipelines and IT infrastructures that are improving over time, is strongly linked to the availability of pre-existing data stored in online databases (e.g. ENA - www.ebi.ac.uk/ena; and NCBI - https://www.ncbi.nlm.nih.gov/).

In this context, data visualization represents an effective strategy not only to aggregate and expose the research results, but also to guide advanced scientific investigations [9, 10]). At this moment, reference databases, where molecular and taxonomic data are friendly explorable and regularly updated, exist only for few molecular markers, such as SILVA for 16S and 18S genes [11], BOLD for animals and plants [12] or UNITE for Fungi domain [13]. However, these data resources are not representative of all the genomic and taxonomic diversity collected to date. On the other hand, although GenBank still resumes the majority of genetic data and their related metadata currently available [14, 15, 16], such information is not always easy to access without specific bioinformatics and IT skills, which constitute a limiting factor to a large audience of scientists.

With the aim to help biologists to improve their experimental designs and to promote data exploration and exploitation, we have developed a tool, ExTaxsI (Exploring Taxonomy Information), able to facilitate the molecular data integration with its associated taxonomy and metadata, eventually retrieved from heterogeneous sources. Moreover, its easy to use interface would greatly help researchers and practitioners in the visualization of either query results obtained from NCBI Nucleotide database (molecular sequences and their metadata) or external user-defined data based on standard taxonomy notation.

To our knowledge, tools that provide user-friendly instruments to download and explore taxonomic data from NCBI have not been completely implemented yet. Currently, there are only a few tools that perform partially this task, focusing on slightly different goals. For example, NCBImeta [17] allows querying NCBI databases via command line scripts, favoring in particular the exploration of metadata associated with the records, but it does not integrate scripts or libraries to promote data visualization and exploration, neither incorporates NCBI taxonomy reference database [18]. On the other hand, TaxonTableTools [19] includes workflows to analyse data produced by the user, focusing on DNA metabarcoding common approaches. ExTaxsI, instead, implements NCBI data retrieval, in order to create formatted databases useful for taxonomy assignment methods and explore the results from a taxonomic and molecular point of view. In particular, it is linked to NCBI taxonomy database [18] and ETE toolkit [20], in order to produce standard formats readable by most common software that deal with taxonomic information [21, 22, 23, 24, 25, 26], such as QIIME2 platform [21]. The tool is applicable to any molecular marker, gene name or taxonomic group data, where it is also possible to create non-standard marker genes database usable in metagenomic/metabarcoding taxonomic assignment tools [21]. In addition, thanks to the integration of the NCBI query tool [27], ExTaxsI can reorganize personal datasets in a standardized format to easily describe taxonomic variability and geographic provenance of records.

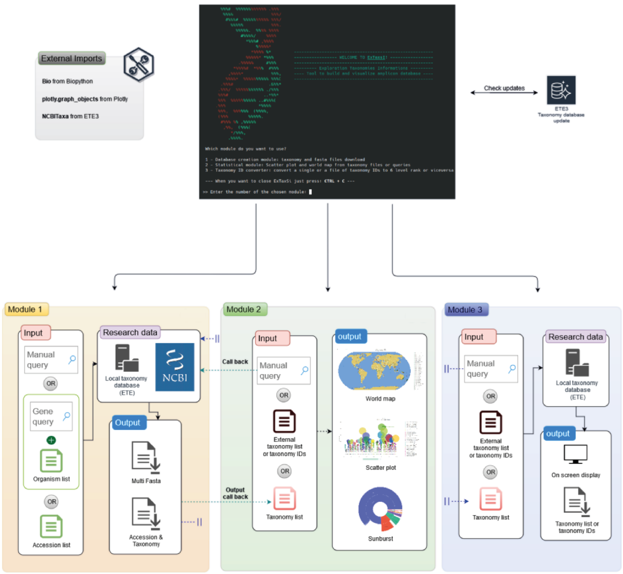

Figure 1. ExTaxsI pipeline: module 1 (orange) searches and creates files and databases; module 2 (green) processes georeferenced or taxonomic data for the creation of graphs and plots; module 3 (blue) converts taxonomic names into NCBI taxonomy ID (TaxID) and vice versa.

ExTaxsI@Work

ExTaxsI is a bioinformatic open-source tool aimed to elaborate and visualize molecular and taxonomic information via a simple interface. It is developed in Python 3.7 both as command line and as a python library. The command line scripts are available through a user-friendly console, as they are built to make the tool interactive, helping the users via questions and explanations. Instead, the Python module was built for IT advanced users to facilitate its integration into specific analytical pipelines (e.g., genomics, metagenomics). As illustrated in Fig. 1, this open-source instrument starting from a list of taxa or gene name/s, allows to i) search for taxonomic, genetic and biogeographical data through NCBI databases, ii) create a local and formatted nucleotide sequences (FASTA format) dataset and iii) their related taxonomy classification paths/datasets, thanks to the integration of NCBI taxonomy data, iv) generate genetic markers lists coming from different studies, and finally v) produce interactive plots starting from NCBI query search results or directly from offline taxonomic files, including representative graphs for the exploration of taxonomy and refinement of biogeographical data by creating geographical maps with the locations of the species analyzed (Figure 1). It is important to note that ExTaxsI outputs are compatible with other tools for taxonomic assignment purposes [22, 23, 24, 25, 26], such as the QIIME2 platform [21].

The communication with NCBI server is mediated by the Entrez module [27], implemented in Biopython library [28], which allows to search, download and parse query results. To help NCBI interaction, for requests less than 2500, the search key is composed by a single query, otherwise the query will be split into groups of 2500 generating temporary files, which are then merged into a single output file at the end of the process.

Regarding taxonomy handling, the ETE toolkit was exploited [20]. In particular, ETE allows to create and maintain a local taxonomy database up to date by extrapolating the 6 main ranks (phylum, class, order, family, genus, and species). If the organism is poorly described or it is an unknown species, the NCBI taxonomy ID (i.e. TaxID) of its ancestor (known as parent TaxID) in ETE taxonomic tree is then used and converted into its scientific correspondent name. It is important to underline

that all queries are carried out locally, avoiding unnecessary online response delays. Finally, the extracted data are visualized through scatter plot and interactive sunburst chart for the taxonomy exploration, and world map plot for the geographic metadata plotting.

Use cases

Being a taxonomy focused data exploration tool, we designed three possible scenarios of variable complexity, to challenge it with increasing taxonomic variability and dimension of accession entries. The first scenario hypothesizes a query to explore data with i) low taxonomic variability and a high number of expected entries (1 species, more than 300,000 entries). The second scenario provides ii) a high taxonomic variability and a large expected number of entries (about 500 species, more than 300,000 entries). The third and more complex scenario explores a iii) complete case study with taxonomic input intersected by molecular data. Considering the case studies of the first two scenarios, we focused on taxa of interest in marine fisheries: 1) the cod fish species (*Gadus morhua*), for which a worldwide economic interest exists, and 2) its taxonomic group at order level – the Gadiformes order – which supports long-standing commercial fisheries and aquaculture. These two case studies evaluate the capacity to explore data and to fill in the geographic distribution of species, prospecting also the available genes information to perform a genetic survey (e.g. in a potential DNA metabarcoding study).

With the third use case, we aimed at demonstrating the flexibility of ExTaxsI in different contexts: a genetic exploration of the available data in NCBI associated to *SARS-CoV-2* virus – a very recent topic that involved many research groups, leading to huge amounts of data collected and deposited in public repositories [29]. A large-scale exploration of data related to this topic can potentially improve the reliability of the results and can provide valuable evidence to inform decisions on public health protection, both now and most importantly in the future.

Insights into two taxonomic groups of commercial interest

The first scenario is the case of *Gadus morhua* species (family: Gadidae; order: Gadiformes), also called Atlantic cod. In details, *Gadus morhua* is a large, cold-adapted teleost fish that supports long-standing commercial fisheries and aquaculture [30, 31, 32, 33, 34].

ExTaxsI retrieved a total of 367,455 accessions (June 18, 2021) using the Taxonomy ID through the following query: "txid8049[ORGN]" (where 8049 is the *Gadus morhua* NCBI TaxID). Only 54,061 entries showed a 'gene' tag investigable by ExTaxsI. As a unique species, we decided to represent the results obtained from a gene survey (Figure 2) and the world map plot (Figure 3).

Regarding gene distribution, the most abundant gene is CYTB – cytochrome b – (with 985 accessions), followed by COI – cytochrome c oxidase subunit I – (455) and ND2 (311). These results are in line with those obtained by Knudsen and colleagues (2019), where they personally developed specific primers for CYTB amplification, as it is a widely used marker in fish molecular characterization. The remaining most abundant genes are the other ND portions and Cytochrome Oxidase fragments (COIII and COII), belonging to the mitochondrial genome. These results show the pronounced effort in sequencing "standard" DNA barcoding markers, while moderately sequencing larger portions of mitochondrial genomes. The remaining genes in the retrieved list and their relative accession frequency distribution (see the complete list in Additional file 1) demonstrate that many regions of the genome were investigated.

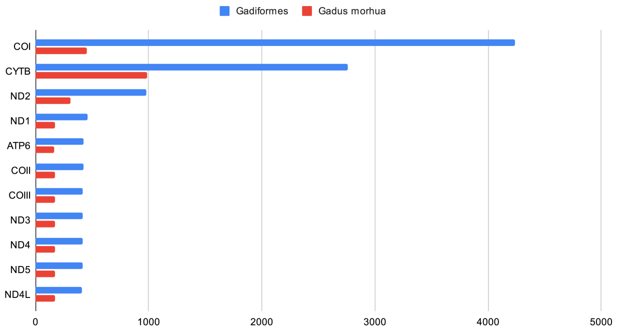

Figure 2. Gene distribution of accessions with available 'gene' tag information among *Gadus morhua* and Gadiformes taxons.

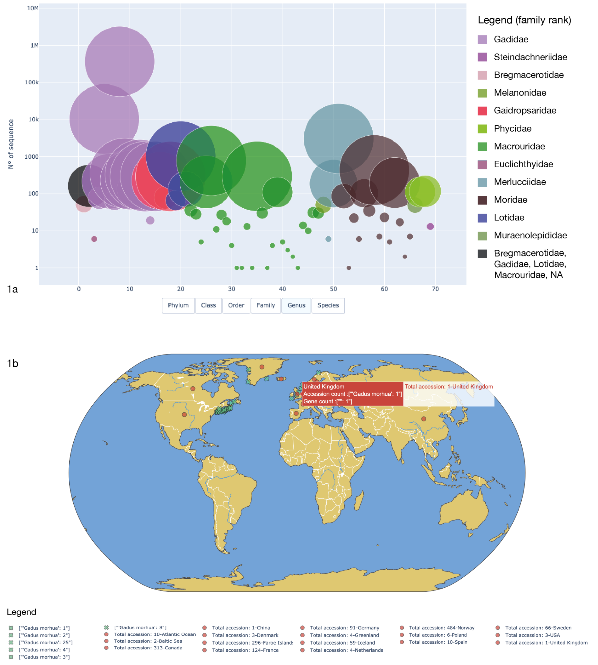

Figure 3. 3a) Scatter plot of Gadiformes accessions representing sequence abundances among families; 3b) World map plot of *Gadus morhua* distribution considering geographic metadata extracted from the records.

Regarding the geographic area, the Gadidae family has a circumpolar distribution, comprising species occurring principally in northern and cool seas [30]. Further, as reported by Jorde and colleagues (2018), in Norway we can recognize four distinct stocks of the Atlantic cod: (1) the oceanic Northeast Arctic cod, (2) coastal cod north of 62°N, (3) coastal cod south of 62°N, and (4) a North Sea/Skagerrak stock, the most densely populated region in Norway [30]. This geographic distribution is partly visible via the metadata extracted by ExTaxsI, as shown in the world map plot in Figure 3b (Additional file 2).

The second scenario takes as an example the Gadiformes Order (phylum: Chordata; class: Actinopterygii), a major group of organisms belonging to marine fisheries. It includes many important food fishes, variously marketed as cods, hakes, grenadiers, moras, moray cods, pelagic cods, codlets and eucla cods [35]. As a vast group, it comprises more than 500 species, which contribute to more than a quarter of the world's marine fish catch [35, 36].

Via ExTaxsI, this order was explored using the following query "txid8043[ORGN]", yielding 389,640 accessions (where

8043 is the specific Gadiformes NCBI TaxID; June 21st, 2021), where 61,249 showed the 'gene' tag information. As a group spread on different taxonomic levels, both taxonomy and gene lists were created. In details, in order to explore taxa distribution and accessions abundances across the entire order, the tool created scatter plot and sunburst plot in HTML format. In Figure 3a genera across families is documented in scatter plot modality, while sunburst plot and entirely interactive plots showing the complete dataset are available in the Supplementary Material section (Additional files 3 and 4).

As shown in Figure 3a, Gadidae is the most abundant family represented by 381,460 accessions, followed by Merlucciidae (3,252) and Macrouridae (1,673). These results are in accordance with the literature, as Gadidae family is a primary marine, bottom-dwelling family of fishes in the Gadiformes order with great commercial power [35, 31].

Further, considering the scatter plot in Additional file 3, the interactive visualization allowed to visualize the taxonomy distribution among the accessions available, changing dynamically the rank to explore. This feature permitted to disclose that the genus Gadus is the most abundant of the entire dataset, in which 94.3% of the accessions corresponded to *Gadus morhua* species. This is an expected result, as *Gadus morhua* is documented to be a key species both in the North Atlantic ecosystem and commercial fisheries, with an increasing aquaculture production in several countries [30].

Considering the genetic information reached by ExTaxsI, a total of 28,850 unique genes were found from the 61,249 completely tagged accessions. A representation of the most ten abundant genes is reported in Figure 2, where at the first position COI gene is placed, a widely used marker gene in DNA metabarcoding projects [31], dealing mainly with animal species identification [1], followed by CYTB and ND2 [1].

Finally, these two case studies showed the ability of the tool to accurately portrait the state of the art of the genetic information available in NCBI. Comparing the most abundant genes found among the records, it is possible to see a thin discrepancy between the two taxa explored (Figure 2), highlighting the disclosures that the survey can report. In general, the detection of mitochondrial genes, coding for COI and CYTB, is in accordance with the reliability of these DNA barcodes, principally used in the discrimination of animal species [37, 38, 39]. To date, considering the subjects of our use cases, different studies have used COI or CYTB barcoding to identify seafood products and explore broad patterns in fish mislabelling [40, 41, 42, 43, 44, 45, 46].

In addition, these use cases higlighted the importance of extracting the geographical metadata from NCBI records. The completeness and the collection of such data can improve drastically the biogeographic and ecological research, allowing not only to explore sampling areas, but also to improve phylogeography investigations, biodiversity monitoring and environmental genomics strategies [1, 47]. Moreover, the retrieved data showed an unbalance between the number of records and the number of explorable genes, which is in some cases due to the incompleteness of the 'gene' tag. In the very recent years, genome sequences started to play a key role in public repositories, making sequences available for sharing and reuse. Submission process can be challenging and errors can affect the availability and the quality of the data. For this reason, there is a wide interest to integrate standardized procedures into the annotation process [48] that can be enhanced by adopting FAIR principles and best practices to avoid the error propagation in sequence databases [49, 50], making the data fully explorable in the future.

Explore biodiversity data in pandemic outbreak: the case of SARS-CoV-2

The severe acute respiratory syndrome coronavirus 2 (*SARS-CoV-2*) is an enveloped, positive-sense, single-stranded RNA virus that causes coronavirus disease 2019 (COVID-19). RNA and structural proteins are included into virus particles mediating host cell invasion. After cell infection, RNA encodes structural proteins that make up virus particles. Virus assembly, transcription, replication and host control are mediated by nonstructural proteins [51]. The pandemic linked to SARS-CoV-2 highlighted hidden virus reservoirs in wild animals and their potential to occasionally spillover into human populations [51]. A detailed understanding of this process is crucial to prevent future spillover events. As reported in the seminal paper of Andersen and colleagues (2020) [52], the risk of future re-emergence events increases if SARS-CoV-2 pre-adapted in another animal species. SARS-CoV-2 probably originated from *Rhinolophus affinis* bats, with pangolin (*Manis javanica*) as intermediate host [52]. Recently, other animal species were supposed to be possible intermediate hosts between bats and humans (Liu et al., 2020; Zhou and Shi, 2021). To date, ACE2 (Angiotensin-converting enzyme 2), the receptor which binds to the receptor-binding domain (RBD) of SARS-CoV-2 S protein [53], is reported as crucial in host invasion.

To test our approach and explore the genetic information available in NCBI, we decided to extrapolate information of the ACE2 gene from the Vertebrata taxonomic group, with the following query: "txid7742[ORGN] AND ACE2[gene]" (where 7742 is the specific Vertebrata NCBI TaxID). The results show that the ACE2 gene is widely distributed throughout Vertebrata as we obtained a total of 1,391 accessions (June 20, 2021), distributed mainly among the Mammalian Class, with a high representation in Actinopteri and Aves groups (Figure 4a; Additional files 5 and 6 for an interactive exploration). In details, Chiroptera, Primates and Rodentia orders are the most represented, with 126, 125 and 81 accessions respectively. In support of this molecular data survey, Luan and colleague (2020) [54] analyzed the affinity of the 20 key amino acid residues in ACE2 to S protein from mammal, bird, turtle, and snake, and suggested that Bovidae (class: Mammalia) and Cricetidae (order: Rodentia) families should be included in the screening of intermediate hosts for *SARS-CoV-2*. In addition, thanks to the analysis of spike glycoprotein sequences from different animals, the study of Dabravolski and Kavalionak (2020) [55] suggested that the human SARS-CoV-2 could also come from yak (family: Bovidae) as an intermediate host. In this context, ExTaxsI has the advantage to provide the complete list of taxa, allowing an exhaustive exploratory research by downloading all the sequences available for the query input, generating in turn the input for downstream analyses, such as the calculation of sequence similarities among different taxa. Further, investigating shared features with other species can have important implications for understanding potential natural reservoirs, zoonotic transmission, and human-to-animal transmission. Noteworthy, the survey can give researchers an instrument to download specific data related to Covid-19, with a user-friendly approach, to explore interactively the data, including biodiversity related information, and to design informed scientific experiments.

Lastly, we explored the data available for *SARS-CoV-2* (Figure 4) using the following query "txid2697049" (where 2697049 is the specific Severe Acute Respiratory Syndrome Coronavirus 2 NCBI TaxID). Figure 4c shows the top ten most abundant genes found in the retrieved entries and corresponding to a total of 773,293 accessions (June 28, 2021). In particular, the most represented genes are: S (59,506) the spike or surface glycoprotein fragment, ORF1AB (58,872), followed by M (58,867), ORF3A (58,867) and N fragments (58,865) the nu-

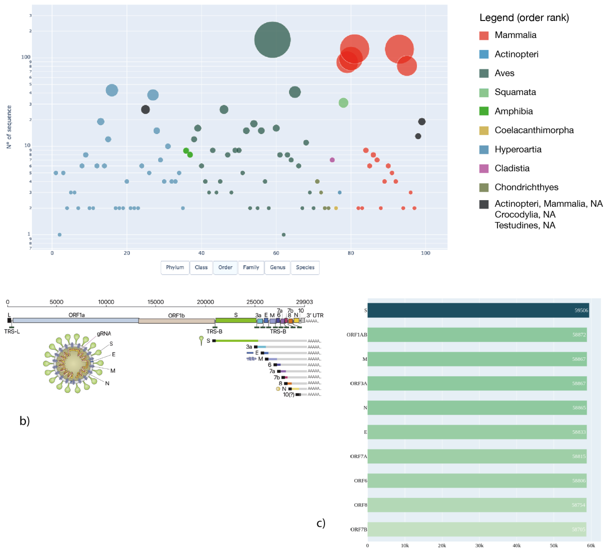

Figure 4. 4a) Scatter plot of ACE2 accessions representing sequence abundances among taxa at order level; 4b) *SARS-CoV-2* representation, from [63]; 4c) gene distribution across accessions of *SARS-CoV-2* data.

cleocapsid protein. These results are in line with the recently published scientific data highlighting the functional aspects of viral proteins. Considering the ORF1AB, several studies demonstrated its pivotal role among coronaviruses [56], providing a clinical target to break down *SARS-CoV-2* infection [57]. In addition, the nucleocapsid phosphoprotein is involved in packaging the RNA into virus particles and protects the viral genome. For these reasons, it has been widely studied and suggested as an antiviral drug target [58, 59]. The spike glycoprotein, instead, is located outside the virus particle, mediating its attachment and promoting the entry into the host cell. It also gives viruses their crown-like appearance. In the very last research, the S protein was found as an important target for diagnostic antigen-based tests, antibody therapies and vaccine development [60, 61]. The entry of *SARS-CoV-2* into host cells is mediated by further processes, for example the activity of the protease TMPRSS2 [62]. Also in this case, the use of ExTaxsI can unearth similar proteases in possible intermediate hosts, revealing new insights into the mechanism of infection.

As also documented in Khailany et al., 2020 [57], the emergent and huge amounts of data collected in the last few months necessitates a large scale exploration. The rapid increment of data releases may give some important insights about *SARS-CoV-2* behaviour in its host species, helping to improve not only our knowledge, but also the design of appropriate prediction models of COVID-19 outbreaks and new target drugs.

Conclusions and future directions

ExTaxsI provides an easy-to-use standalone tool able to interact with NCBI databases and personal datasets, offering instruments to standardize taxonomy information and visualize vast amount of data distributed on different taxonomic levels. It also provides interactive visualization plots, easily shareable through HTML formats.

The user-oriented interrogation of NCBI databases may help researchers involved in environmental genomics fields, from phylogeographic studies to DNA metabarcoding surveys, and also in projects related to human health, as demonstrated with the *SARS-CoV-2* case study.

With this work, we hope to meet the needs of a broad group of researchers, providing an instrument easy to install either on common laptops or on high performance servers and directly connected with NCBI databases. In parallel to the command-line tool, a python library containing all ExTaxsI functions has been implemented, favoring a direct incorporation of such functions into data analysis and exploration pipelines.

In addition, as data volume is increasing over time and NCBI databases still have a few constraints regarding the queries results dimension and their retrieval time required, an automatic management of large queries will be organized in future releases. Finally, we will also consider further data visualization strategies and additional metadata (e.g. GBIF country information) to enhance data interpretation and to provide comprehensive sets of relevant scientific-focused information. In our opinion, ExTaxsI data management ability with its visual interactive exploration can really improve the experimental design phase and the awareness of the information available, facilitating data examination and sharing.

Implementation

ExTaxsI is a bioinformatic tool aimed to explore, elaborate and visualize molecular and taxonomic information via a simple user interface without specific bioinformatic or programming skills. The tool can be run, via command line interface, where the user is guided by the appropriate documentation of each script, avoiding the implementation of ad hoc python code. ExTaxsI is developed in three separate modules, which can be used either interconnected as workflow or independent according to the user needs. The main modules are listed as follows: i) Database creation, ii) Visualization and iii) Taxonomy ID converter.

ExTaxsI is also available as a python library that can be installed through `pip` (package installer for Python), containing the same functions and parameters as those of the command-line tool. A detailed description of each module is provided below.

1 - Database creation module

The module 'Database' allows the user to create multi FASTA files composed of nucleotide sequences, taxonomic lists, genes names and their related accessions, starting from either a single or a batch query mode using csv/tsv input files (Figure 1). After indicating the input type, it is possible to integrate the query with one or more gene name/s (or other details). This step allows to restrict the search in NCBI databases if needed. In general, the output formats are i) a multi-FASTA file (widely used format for molecular sequences) and ii) text file in TSV format, with two columns composed by the accessions code followed by the taxonomy path of each accession at the six main levels separated by semicolons: phylum, class, order, family, genus and species. When requested by the user, the output file of genes names is provided in TSV format consisting of a table with two columns, the first is the list of genes and the other is the frequency values of the respective genes found in the retrieved records. The tool also provides a summary table containing the most popular genes from a list of NCBI taxids, accessions or organisms. In addition, it is possible to create a barplot with the top ten of the summary table, downloadable as a PNG file.

2 - Visualization module

The module 'Visualization' allows the user to create interactive plots, starting from the 'Database' module output or from external sources such as local files (e.g., Additional files 3, 4 and 5) containing taxonomic lists. Before producing the plots, a dialogue box will ask the user to choose a filter value on the data based on the frequency. If the chosen filter value is 0, the tool processes all the data. Otherwise, all the taxonomic units that have not reached the minimum value are inserted into an additional text file, specifically created with a name containing the filter used.

The available plots generated by ExTaxsI are i) scatter plot (Additional file 3), ii) sunburst plot (Additional file 4) and iii) world map plot (Additional file 2). All figures created by the Visualization module can be downloaded as HTML format files. In details, scatter plot uses taxonomy as input to produce a graph that indicates the quantity of each individual taxonomic unit; the interactive plot enables the user to i) choose the taxonomic level to be displayed using the buttons located under the graph and ii) hover over points to show details, such as the number of records within taxa, names of selected taxa and name of the parent taxon. The plot allows also to compare more data on mouse-over, highlight an area of interest with zoom function and view of a specific group or remove specific taxa from the graph. Sunburst plot, instead, from a taxonomy input creates an expansion pie that allows exploring taxonomy by clicking on the taxonomic group of interest and showing the underlying taxa within a new sunburst plot. Also in this case, hovering over points shows the number of records within taxa. Regarding world map plot, the initial input is processed in order to obtain geographic data. The tool exploits the 'Country' metadata stored in the NCBI records to produce a map indicating the position of each entry. In this step, based on the type of geographic data obtained, ExTaxsI divides results into two different arrays: i) a specific array of coordinates (if the coordinates are present in the record) or ii) a specific array of country names (if the coordinates are absent). It is also possible to add external sources data to the map. In each created map, the coordinates are indicated by green X signs, while countries by red circles. Thinking of multiple taxa plotting, each symbol can have a legend that summarizes the data downloaded with the same country name or coordinates description. Further, it is possible to see both genes and counts available among the represented accessions.

3 - Taxonomy ID converter module

This module allows to convert NCBI TaxID into the main six taxonomy ranks and vice versa (phylum, class, order, family, genus and species); it can convert single manual inputs or multiple inputs from a tsv/csv file containing a list of TaxIDs.

Availability of source code and requirements

Command-line tool

No specific system requirements are needed for the installation of ExTaxsI, however for the correct functioning of the software we suggest a minimum of 4GB of RAM. To successfully run ExTaxsI, the following python libraries must be installed: Biopython [28], NumPy [64]), SciPy [65], Matplotlib [66], ipython [67], Pandas [68], SymPy (https://www.sympy.org/en/index.html), nose (https://nose.readthedocs.io/en/latest/), genutils (https://pypi.org/project/genutils/), requests [69] and Plotly (https://plotly.com/), in addition to Plotly-Orca and ETE toolkit [20]. To install all the dependencies compatible versions, we provide a requirement list at the GitHub page https://github.com/qLSLab/ExTaxsI, with a detailed guideline to set directly a conda environment.

Python library

The Python library `extaxsi` is available both in the Github page: https://github.com/qLSLab/ExTaxsI/tree/master/library and in PyPI repository: https://pypi.org/project/extaxsi/

- Project name: ExTaxsI
- Project home page: https://github.com/qLSLab/extaxsi; https://github.com/qLSLab/ExTaxsI/tree/master/library; https://pypi.org/project/extaxsi/
- Operating system(s): Platform independent
- Programming language: Python
- License: GNU GPL version 3

Availability of supporting data and materials

Additional Files

Additional file 1: Gene list in TSV format obtained through ExTaxsI for the species *Gadus morhua*. Gene counts were extracted from 367,455 accessions (query: "txid8049[ORGN]"; 18 of June, 2021).
Additional file 2: World map plot in HTML format created via ExTaxsI extracting the values of 'Country' tag contained in 367,4553 accessions of *Gadus morhua* (query: "txid8049[ORGN]"; 18 of June, 2021). Coordinates are indicated by green X signs, while States by red circles.
Additional file 3: Scatterplot in HTML format created via ExTaxsI extracting the taxonomy of 389,640 accessions of Gadiformes Order (txid8043[ORGN]"; 21 of June, 2021).
Additional file 4: Sunburst plot in HTML format created via ExTaxsI extracting the taxonomy of 388,603 accessions of Gadiformes order (txid8043[ORGN]"; 21 of June, 2021).
Additional file 5: Scatterplot in HTML format created via ExTaxsI extracting the taxonomy related to 1,391 accessions of ACE2 genes belonging to the Vertebrata taxonomic group (query: "txid7742[ORGN] AND ACE2[gene]"; 20 of June, 2021).
Additional file 6: Sunburst plot in HTML format created via ExTaxsI extracting the taxonomy related to 1,391 accessions of ACE2 genes belonging to the Vertebrata taxonomic group (query: "txid7742[ORGN] AND ACE2[gene]"; 20 of June, 2021).

Declarations

List of abbreviations

SILVA: High quality ribosomal RNA databases; BOLD: Barcode of Life Data System; UNITE: Database and sequence management environment centered on the eukaryotic nuclear ribosomal ITS region; ETE: Environment for Tree Exploration; QIIME2: Quantitative Insights Into Microbial Ecology; FASTA: Text-based format for representing either nucleotide sequences or peptide sequences; TAXID: Taxonomy ID; HTML: Hyper-Text Markup Language; COI: Cytochrome Oxidase I; COI: Cytochrome Oxidase II; COIII: Cytochrome Oxidase III; CYTB: Cytochrome B; ND2: NADH dehydrogenase 2; ACE2: Angiotensin-Converting enzyme 2; RBD: Receptor-Binding Domain; PNG: Portable Network Graphics; NCBI: National Center for Biotechnology Information; ENA: European Nucleotide Archive

Consent for publication

Not applicable.

Competing Interests

The authors declare that they have no competing interests.

Funding

This study was funded by the 'Ministero dell'Istruzione dell'Università e della Ricerca' (MIUR) within the project: 'Sistemi Alimentari e Sviluppo Sostenibile—tra ricerca e processi internazionali e africani'. CUP: H42F16002450001. The funder had no role in conducting the research and/or during the preparation of the article.

Author's Contributions

Giulia Agostinetto: Conceptualization, Investigation, Software development, Visualization, Original Draft Preparation, Review, Editing, Supervision, Project Administration. **Alberto Brusati**: Investigation, Software development, Visualization, Review & Editing. **Anna Sandionigi**: Conceptualization, Original Draft Preparation, Review & Editing, Supervision, Project Administration. **Adam Chahed**: Software development, Visualization. **Elena Parladori**: Software development, Visualization. **Bachir Balech**: Review & Editing, Validation. **Antonia Bruno**: Review & Editing, Validation. **Dario Pescini**: Review & Editing, Supervision. **Maurizio Casiraghi**: Funding Acquisition, Supervision. All authors read and approved the final manuscript, contributing critically important comments.

Acknowledgements

The authors thank all the ELIXIR Biodiversity community members for the support and all researchers who have provided input on the development of ExTaxsI project.

References

1. Porter TM, Hajibabaei M. Scaling up: A guide to high-throughput genomic approaches for biodiversity analysis. Molecular ecology 2018;27(2):313–338.
2. Ruppert KM, Kline RJ, Rahman MS. Past, present, and future perspectives of environmental DNA (eDNA) metabarcoding: A systematic review in methods, monitoring, and applications of global eDNA. Global Ecology and Conservation 2019;17:e00547.
3. Deiner K, Bik HM, Mächler E, Seymour M, Lacoursière-Roussel A, Altermatt F, et al. Environmental DNA metabarcoding: Transforming how we survey animal and plant communities. Molecular ecology 2017;26(21):5872–5895.
4. Hampton SE, Jones MB, Wasser LA, Schildhauer MP, Supp SR, Brun J, et al. Skills and knowledge for data-intensive environmental research. BioScience 2017;67(6):546–557.
5. White EP, Baldridge E, Brym ZT, Locey KJ, McGlinn DJ, Supp SR. Nine simple ways to make it easier to (re) use your data. Ideas in Ecology and Evolution 2013;6(2).
6. Michener WK, Jones MB. Ecoinformatics: supporting ecology as a data-intensive science. Trends in ecology & evolution 2012;27(2):85–93.
7. Mitchell AL, Almeida A, Beracochea M, Boland M, Burgin J, Cochrane G, et al. MGnify: the microbiome analysis resource in 2020. Nucleic acids research 2020;48(D1):D570–D578.
8. Almeida A, Mitchell AL, Boland M, Forster SC, Gloor GB, Tarkowska A, et al. A new genomic blueprint of the human gut microbiota. Nature 2019;568(7753):499–504.
9. Kaur P, Klan F, König-Ries B. Issues and Suggestions for the Development of a Biodiversity Data Visualization Support Tool. In: EuroVis (Short Papers); 2018. p. 73–77.
10. Hardisty A, Roberts D, et al. A decadal view of biodiversity informatics: challenges and priorities. BMC ecology 2013;13(1):16.
11. Pruesse E, Quast C, Knittel K, Fuchs BM, Ludwig W, Peplies J, et al. SILVA: a comprehensive online resource for quality checked and aligned ribosomal RNA sequence data compatible with ARB. Nucleic acids research 2007;35(21):7188–7196.
12. Ratnasingham S, Hebert PD. BOLD: The Barcode of Life Data System (http://www. barcodinglife. org). Molecular ecology notes 2007;7(3):355–364.
13. Nilsson RH, Larsson KH, Taylor AFS, Bengtsson-Palme J, Jeppesen TS, Schigel D, et al. The UNITE database for molecular identification of fungi: handling dark taxa and parallel taxonomic classifications. Nucleic acids research 2019;47(D1):D259–D264.
14. Keller A, Hohlfeld S, Kolter A, Schultz J, Gemeinholzer B, Ankenbrand MJ. BCdatabaser: on-the-fly reference database creation for (meta-) barcoding. Bioinformatics 2020;36(8):2630–2631.
15. Ankenbrand MJ, Keller A, Wolf M, Schultz J, Förster F. ITS2 database V: Twice as much. Molecular Biology and Evolution 2015;32(11):3030–3032.
16. Benson D, Karsch-Mizrachi I, Lipman D, Ostell J, Wheeler D. GenBank Nucleic Acids Res. jan 2008;1:33.
17. Eaton K. NCBImeta: Efficient and comprehensive metadata retrieval from NCBI databases. Journal of Open Source Software 2020;5(46):1990.
18. Federhen S. The NCBI taxonomy database. Nucleic acids research 2012;40(D1):D136–D143.
19. Macher TH, Beermann AJ, Leese F. TaxonTableTools: A comprehensive, platform-independent graphical user interface software to explore and visualise DNA metabarcoding data. Molecular Ecology Resources 2021;.
20. Huerta-Cepas J, Serra F, Bork P. ETE 3: reconstruction, analysis, and visualization of phylogenomic data. Molecular biology and evolution 2016;33(6):1635–1638.
21. Bolyen E, Rideout JR, Dillon MR, Bokulich NA, Abnet CC, Al-Ghalith GA, et al. Reproducible, interactive, scalable and extensible microbiome data science using QIIME 2. Nature biotechnology 2019;37(8):852–857.
22. Rognes T, Flouri T, Nichols B, Quince C, Mahé F. VSEARCH: a versatile open source tool for metagenomics. PeerJ 2016;4:e2584.
23. Bengtsson-Palme J, Hartmann M, Eriksson KM, Pal C, Thorell K, Larsson DGJ, et al. METAXA2: improved identification and taxonomic classification of small and large subunit rRNA in metagenomic data. Molecular ecology resources 2015;15(6):1403–1414.
24. Mahé F, Rognes T, Quince C, de Vargas C, Dunthorn M. Swarm v2: highly-scalable and high-resolution amplicon clustering. PeerJ 2015;3:e1420.
25. Camacho C, Coulouris G, Avagyan V, Ma N, Papadopoulos J, Bealer K, et al. BLAST+: architecture and applications. BMC bioinformatics 2009;10(1):421.
26. Wang Q, Garrity GM, Tiedje JM, Cole JR. Naive Bayesian classifier for rapid assignment of rRNA sequences into the new bacterial taxonomy. Applied and environmental microbiology 2007;73(16):5261–5267.
27. Coordinators NR. Database resources of the national cen-

ter for biotechnology information. Nucleic acids research 2014;42(D1):D7–D17.
28. Cock PJ, Antao T, Chang JT, Chapman BA, Cox CJ, Dalke A, et al. Biopython: freely available Python tools for computational molecular biology and bioinformatics. Bioinformatics 2009;25(11):1422–1423.
29. Blomberg N, Lauer KB. Connecting data, tools and people across Europe: ELIXIR's response to the COVID-19 pandemic. European Journal of Human Genetics 2020;p. 1–5.
30. Jorde PE, Kleiven AR, Sodeland M, Olsen EM, Ferter K, Jentoft S, et al. Who is fishing on what stock: population-of-origin of individual cod (Gadus morhua) in commercial and recreational fisheries. ICES Journal of Marine Science 2018;75(6):2153–2162.
31. Knudsen SW, Ebert RB, Hesselsøe M, Kuntke F, Hassingboe J, Mortensen PB, et al. Species-specific detection and quantification of environmental DNA from marine fishes in the Baltic Sea. Journal of experimental marine biology and ecology 2019;510:31–45.
32. Star B, Nederbragt AJ, Jentoft S, Grimholt U, Malmstrøm M, Gregers TF, et al. The genome sequence of Atlantic cod reveals a unique immune system. Nature 2011;477(7363):207–210.
33. Kurlansky M, Davidson RM. Cod: a Biography of the Fish that Changed the world. Phoenix Books; 2006.
34. Johansen SD, Coucheron DH, Andreassen M, Karlsen BO, Furmanek T, Jørgensen TE, et al. Large-scale sequence analyses of Atlantic cod. New Biotechnology 2009;25(5):263–271.
35. Nelson JS, Grande TC, Wilson MV. Fishes of the World. John Wiley & Sons; 2016.
36. Costello MJ, Bouchet P, Boxshall G, Fauchald K, Gordon D, Hoeksema BW, et al. Global coordination and standardisation in marine biodiversity through the World Register of Marine Species (WoRMS) and related databases. PloS one 2013;8(1):e51629.
37. Hebert PD, Ratnasingham S, De Waard JR. Barcoding animal life: cytochrome c oxidase subunit 1 divergences among closely related species. Proceedings of the Royal Society of London Series B: Biological Sciences 2003;270(suppl_1):S96–S99.
38. Hellberg RS, Kawalek MD, Van KT, Shen Y, Williams-Hill DM. Comparison of DNA extraction and PCR setup methods for use in high-throughput DNA barcoding of fish species. Food analytical methods 2014;7(10):1950–1959.
39. Mueller S, Handy SM, Deeds JR, George GO, Broadhead WJ, Pugh SE, et al. Development of a COX1 based PCR-RFLP method for fish species identification. Food Control 2015;55:39–42.
40. Fernandes TJ, Costa J, Oliveira MBP, Mafra I. DNA barcoding coupled to HRM analysis as a new and simple tool for the authentication of Gadidae fish species. Food Chemistry 2017;230:49–57.
41. Cline E. Marketplace substitution of Atlantic salmon for Pacific salmon in Washington State detected by DNA barcoding. Food Research International 2012;45(1):388–393.
42. Di Pinto A, Di Pinto P, Terio V, Bozzo G, Bonerba E, Ceci E, et al. DNA barcoding for detecting market substitution in salted cod fillets and battered cod chunks. Food chemistry 2013;141(3):1757–1762.
43. Miller DD, Mariani S. Smoke, mirrors, and mislabeled cod: poor transparency in the European seafood industry. Frontiers in Ecology and the Environment 2010;8(10):517–521.
44. Rasmussen RS, Morrissey MT. DNA-based methods for the identification of commercial fish and seafood species. Comprehensive reviews in food science and food safety 2008;7(3):280–295.
45. Wong EHK, Hanner RH. DNA barcoding detects market substitution in North American seafood. Food Research International 2008;41(8):828–837.
46. Yancy HF, Zemlak TS, Mason JA, Washington JD, Tenge BJ, Nguyen NLT, et al. Potential use of DNA barcodes in regulatory science: applications of the Regulatory Fish Encyclopedia. Journal of Food Protection 2008;71(1):210–217.
47. Cordier T, Alonso-Sáez L, Apothéloz-Perret-Gentil L, Aylagas E, Bohan DA, Bouchez A, et al. Ecosystems monitoring powered by environmental genomics: a review of current strategies with an implementation roadmap. Molecular Ecology 2020;.
48. Geib SM, Hall B, Derego T, Bremer FT, Cannoles K, Sim SB. Genome Annotation Generator: a simple tool for generating and correcting WGS annotation tables for NCBI submission. GigaScience 2018;7(4):giy018.
49. Wilkinson MD, Dumontier M, Aalbersberg IJ, Appleton G, Axton M, Baak A, et al. The FAIR Guiding Principles for scientific data management and stewardship. Scientific data 2016;3(1):1–9.
50. Pirovano W, Boetzer M, Derks MF, Smit S. NCBI-compliant genome submissions: tips and tricks to save time and money. Briefings in Bioinformatics 2017;18(2):179–182.
51. Lu R, Zhao X, Li J, Niu P, Yang B, Wu H, et al. Genomic characterisation and epidemiology of 2019 novel coronavirus: implications for virus origins and receptor binding. The Lancet 2020;395(10224):565–574.
52. Andersen KG, Rambaut A, Lipkin WI, Holmes EC, Garry RF. The proximal origin of SARS-CoV-2. Nature medicine 2020;26(4):450–452.
53. Letko M, Marzi A, Munster V. Functional assessment of cell entry and receptor usage for SARS-CoV-2 and other lineage B betacoronaviruses. Nature microbiology 2020;5(4):562–569.
54. Luan J, Jin X, Lu Y, Zhang L. SARS-CoV-2 spike protein favors ACE2 from Bovidae and Cricetidae. Journal of medical virology 2020;.
55. Dabravolski SA, Kavalionak YK. SARS-CoV-2: Structural diversity, phylogeny, and potential animal host identification of spike glycoprotein. Journal of medical virology 2020;.
56. Wan Y, Shang J, Graham R, Baric RS, Li F. Receptor recognition by the novel coronavirus from Wuhan: an analysis based on decade-long structural studies of SARS coronavirus. Journal of virology 2020;94(7).
57. Khailany RA, Safdar M, Ozaslan M. Genomic characterization of a novel SARS-CoV-2. Gene reports 2020;p. 100682.
58. Wu F, Zhao S, Yu B, Chen YM, Wang W, Song ZG, et al. A new coronavirus associated with human respiratory disease in China. Nature 2020;579(7798):265–269.
59. Gordon DE, Jang GM, Bouhaddou M, Xu J, Obernier K, White KM, et al. A SARS-CoV-2 protein interaction map reveals targets for drug repurposing. Nature 2020;p. 1–13.
60. Salvatori G, Luberto L, Maffei M, Aurisicchio L, Roscilli G, Palombo F, et al. SARS-CoV-2 SPIKE PROTEIN: an optimal immunological target for vaccines. Journal of Translational Medicine 2020;18:1–3.
61. Pillay TS. Gene of the month: the 2019-nCoV/SARS-CoV-2 novel coronavirus spike protein. Journal of Clinical Pathology 2020;.
62. Hoffmann M, Kleine-Weber H, Schroeder S, Krüger N, Herrler T, Erichsen S, et al. SARS-CoV-2 cell entry depends on ACE2 and TMPRSS2 and is blocked by a clinically proven protease inhibitor. Cell 2020;.
63. Kim D, Lee JY, Yang JS, Kim JW, Kim VN, Chang H. The architecture of SARS-CoV-2 transcriptome. Cell 2020;.
64. Harris CR, Millman KJ, van der Walt SJ, Gommers R, Virtanen P, Cournapeau D, et al. Array programming with

NumPy. Nature 2020;585(7825):357–362.
65. Virtanen P, Gommers R, Oliphant TE, Haberland M, Reddy T, Cournapeau D, et al. SciPy 1.0: fundamental algorithms for scientific computing in Python. Nature methods 2020;17(3):261–272.
66. Hunter JD. Matplotlib: A 2D graphics environment. Computing in science & engineering 2007;9(03):90–95.
67. Pérez F, Granger BE. IPython: a system for interactive scientific computing. Computing in science & engineering 2007;9(3):21–29.
68. McKinney W, et al. pandas: a foundational Python library for data analysis and statistics. Python for high performance and scientific computing 2011;14(9):1–9.
69. Chandra RV, Varanasi BS. Python requests essentials. Packt Publishing Ltd; 2015.

pdf of the main manuscript
Click here to access/download;Manuscript;Agostinetto_et_al_RESubmission

OXFORD (GIGA)^n SCIENCE

GigaScience, 2017, 1–9

doi: xx.xxxx/xxxx
Manuscript in Preparation
Paper

PAPER

ExTaxsI: an exploration tool of biodiversity molecular data

Giulia Agostinetto[1,*], Alberto Brusati[2,*], Anna Sandionigi[3,†], Adam Chahed[1], Elena Parladori[1], Bachir Balech[4], Antonia Bruno[1], Dario Pescini[5] and Maurizio Casiraghi[1]

[1]University of Milano-Bicocca, Department of Biotechnology and Biosciences, Milan, Italy and [2]Istituto Auxologico Italiano, Milan, Italy and [3]Quantia Consulting srl, Milan, Italy and [4]Institute of Biomembranes, Bioenergetics and Molecular Biotechnologies (CNR), via Amendola 122/O, 70126, Bari, Italy and [5]University of Milano-Bicocca, Department of Statistics and Quantitative Methods, Milan, Italy

*These authors contributed equally to the work.
†corresponding author: anna.sandionigi@quantiaconsulting.com

Abstract

Background The increasing availability of multi-omics data is leading to regularly revise estimates of existing biodiversity data. In particular, the molecular data enable to characterize novel species yet unknown and to increase the information linked to those already observed with new genomics data. For this reason, the management and visualization of existing molecular data, and their related metadata, through the implementation of easy-to-use IT tools have become a key point to design future research. The more users are able to access biodiversity related information, the greater the ability of the scientific community to expand its knowledge in this area. **Results** In this paper we have focused on the development of ExTaxsI (Exploring Taxonomy Information), an IT tool able to retrieve biodiversity data stored in NCBI databases and provide a simple and explorable visualization. Through the three case studies presented here, we have shown how an efficient organization of the available data can lead to obtain new information that is fundamental as a starting point for a new research of interest. Using this approach, it was possible to highlight the limits in the distribution of data availability, a key factor to consider in the experimental design phase of broad spectrum studies such as metagenomics. **Conclusions** ExTaxsI can easily retrieve molecular data and its metadata with an explorable visualization, with the aim to help researchers to improve experimental designs and highlight the main gaps in the coverage of available data.

Key words: Biodiversity; Data visualization; Molecular data; Database; Data integration; Taxonomy gaps

Introduction

In recent years, studies investigating biodiversity at large scale have started to create and incorporate molecular data in biological databases. In particular, the spread of metagenomics studies (e.g. DNA metabarcoding) have contributed to an exponential increase in genomics data availability. Thanks to this large amount of new information it is possible to expand our knowledge and enhance our scientific investigation capacity in many fields of research [1], ranging from macro-ecology and ecosystem monitoring, to food safety control, forensics applications and microbiome identification [2, 1, 3]. Different groups of researchers emphasized the wealth of information collected in biological and molecular databases, with the aim to improve data usefulness and reusability [4, 5, 6]. Therefore, building experimental designs that consider the totality of the data present in

Compiled on: November 16, 2021.
Draft manuscript prepared by the author.

such databases could certainly increase the efficiency of these studies, and lead to more robust results [7, 8].

Biodiversity data retrieval and exploration are listed among the challenges of "big data" science, forcing researchers to use Information Technologies (IT) tools for their management. In particular, the interpretation of results derived from metagenomic experiments, requiring computational pipelines and IT infrastructures that are improving over time, is strongly linked to the availability of pre-existing data stored in online databases (e.g. ENA - www.ebi.ac.uk/ena; and NCBI - https://www.ncbi.nlm.nih.gov/).

In this context, data visualization represents an effective strategy not only to aggregate and expose the research results, but also to guide advanced scientific investigations [9, 10]). At this moment, reference databases, where molecular and taxonomic data are friendly explorable and regularly updated, exist only for few molecular markers, such as SILVA for 16S and 18S genes [11], BOLD for animals and plants [12] or UNITE for Fungi domain [13]. However, these data resources are not representative of all the genomic and taxonomic diversity collected to date. On the other hand, although GenBank still resumes the majority of genetic data and their related metadata currently available [14, 15, 16], such information is not always easy to access without specific bioinformatics and IT skills, which constitute a limiting factor to a large audience of scientists.

With the aim to help biologists to improve their experimental designs and to promote data exploration and exploitation, we have developed a tool, ExTaxsI (Exploring Taxonomy Information), able to facilitate the molecular data integration with its associated taxonomy and metadata, eventually retrieved from heterogeneous sources. Moreover, its easy to use interface would greatly help researchers and practitioners in the visualization of either query results obtained from NCBI Nucleotide database (molecular sequences and their metadata) or external user-defined data based on standard taxonomy notation.

To our knowledge, tools that provide user-friendly instruments to download and explore taxonomic data from NCBI have not been completely implemented yet. Currently, there are only a few tools that perform partially this task, focusing on slightly different goals. For example, NCBImeta [17] allows querying NCBI databases via command line scripts, favoring in particular the exploration of metadata associated with the records, but it does not integrate scripts or libraries to promote data visualization and exploration, neither incorporates NCBI taxonomy reference database [18]. On the other hand, TaxonTableTools [19] includes workflows to analyse data produced by the user, focusing on DNA metabarcoding common approaches. ExTaxsI, instead, implements NCBI data retrieval, in order to create formatted databases useful for taxonomy assignment methods and explore the results from a taxonomic and molecular point of view. In particular, it is linked to NCBI taxonomy database [18] and ETE toolkit [20], in order to produce standard formats readable by most common software that deal with taxonomic information [21, 22, 23, 24, 25, 26], such as QIIME2 platform [21]. The tool is applicable to any molecular marker, gene name or taxonomic group data, where it is also possible to create non-standard marker genes database usable in metagenomic/metabarcoding taxonomic assignment tools [21]. In addition, thanks to the integration of the NCBI query tool [27], ExTaxsI can reorganize personal datasets in a standardized format to easily describe taxonomic variability and geographic provenance of records.

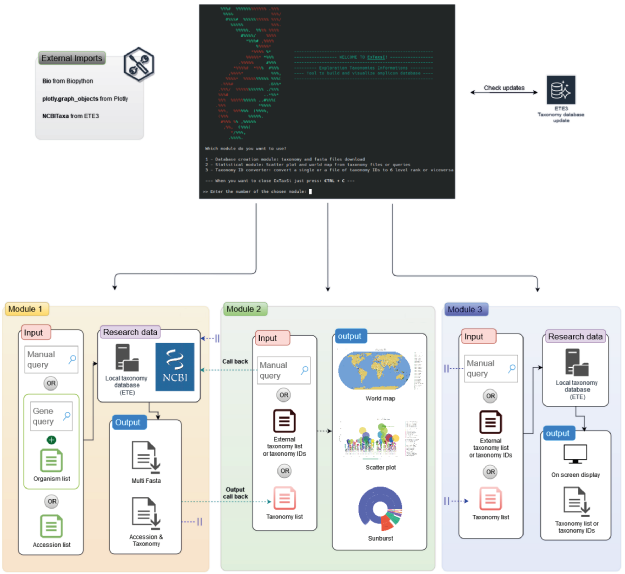

Figure 1. ExTaxsI pipeline: module 1 (orange) searches and creates files and databases; module 2 (green) processes georeferenced or taxonomic data for the creation of graphs and plots; module 3 (blue) converts taxonomic names into NCBI taxonomy ID (TaxID) and vice versa.

ExTaxsI@Work

ExTaxsI is a bioinformatic open-source tool aimed to elaborate and visualize molecular and taxonomic information via a simple interface. It is developed in Python 3.7 both as command line and as a python library. The command line scripts are available through a user-friendly console, as they are built to make the tool interactive, helping the users via questions and explanations. Instead, the Python module was built for IT advanced users to facilitate its integration into specific analytical pipelines (e.g., genomics, metagenomics). As illustrated in Fig. 1, this open-source instrument starting from a list of taxa or gene name/s, allows to i) search for taxonomic, genetic and biogeographical data through NCBI databases, ii) create a local and formatted nucleotide sequences (FASTA format) dataset and iii) their related taxonomy classification paths/datasets, thanks to the integration of NCBI taxonomy data, iv) generate genetic markers lists coming from different studies, and finally v) produce interactive plots starting from NCBI query search results or directly from offline taxonomic files, including representative graphs for the exploration of taxonomy and refinement of biogeographical data by creating geographical maps with the locations of the species analyzed (Figure 1). It is important to note that ExTaxsI outputs are compatible with other tools for taxonomic assignment purposes [22, 23, 24, 25, 26], such as the QIIME2 platform [21].

The communication with NCBI server is mediated by the Entrez module [27], implemented in Biopython library [28], which allows to search, download and parse query results. To help NCBI interaction, for requests less than 2500, the search key is composed by a single query, otherwise the query will be split into groups of 2500 generating temporary files, which are then merged into a single output file at the end of the process.

Regarding taxonomy handling, the ETE toolkit was exploited [20]. In particular, ETE allows to create and maintain a local taxonomy database up to date by extrapolating the 6 main ranks (phylum, class, order, family, genus, and species). If the organism is poorly described or it is an unknown species, the NCBI taxonomy ID (i.e. TaxID) of its ancestor (known as parent TaxID) in ETE taxonomic tree is then used and converted into its scientific correspondent name. It is important to underline

that all queries are carried out locally, avoiding unnecessary online response delays. Finally, the extracted data are visualized through scatter plot and interactive sunburst chart for the taxonomy exploration, and world map plot for the geographic metadata plotting.

Use cases

Being a taxonomy focused data exploration tool, we designed three possible scenarios of variable complexity, to challenge it with increasing taxonomic variability and dimension of accession entries. The first scenario hypothesizes a query to explore data with i) low taxonomic variability and a high number of expected entries (1 species, more than 300,000 entries). The second scenario provides ii) a high taxonomic variability and a large expected number of entries (about 500 species, more than 300,000 entries). The third and more complex scenario explores a iii) complete case study with taxonomic input intersected by molecular data. Considering the case studies of the first two scenarios, we focused on taxa of interest in marine fisheries: 1) the cod fish species (*Gadus morhua*), for which a worldwide economic interest exists, and 2) its taxonomic group at order level – the Gadiformes order – which supports long-standing commercial fisheries and aquaculture. These two case studies evaluate the capacity to explore data and to fill in the geographic distribution of species, prospecting also the available genes information to perform a genetic survey (e.g. in a potential DNA metabarcoding study).

With the third use case, we aimed at demonstrating the flexibility of ExTaxsI in different contexts: a genetic exploration of the available data in NCBI associated to *SARS-CoV-2* virus – a very recent topic that involved many research groups, leading to huge amounts of data collected and deposited in public repositories [29]. A large-scale exploration of data related to this topic can potentially improve the reliability of the results and can provide valuable evidence to inform decisions on public health protection, both now and most importantly in the future.

Insights into two taxonomic groups of commercial interest

The first scenario is the case of *Gadus morhua* species (family: Gadidae; order: Gadiformes), also called Atlantic cod. In details, *Gadus morhua* is a large, cold-adapted teleost fish that supports long-standing commercial fisheries and aquaculture [30, 31, 32, 33, 34].

ExTaxsI retrieved a total of 367,455 accessions (June 18, 2021) using the Taxonomy ID through the following query: “txid8049[ORGN]” (where 8049 is the *Gadus morhua* NCBI TaxID). Only 54,061 entries showed a ‘gene’ tag investigable by ExTaxsI. As a unique species, we decided to represent the results obtained from a gene survey (Figure 2) and the world map plot (Figure 3).

Regarding gene distribution, the most abundant gene is CYTB – cytochrome b – (with 985 accessions), followed by COI – cytochrome c oxidase subunit I – (455) and ND2 (311). These results are in line with those obtained by Knudsen and colleagues (2019), where they personally developed specific primers for CYTB amplification, as it is a widely used marker in fish molecular characterization. The remaining most abundant genes are the other ND portions and Cytochrome Oxidase fragments (COIII and COII), belonging to the mitochondrial genome. These results show the pronounced effort in sequencing "standard" DNA barcoding markers, while moderately sequencing larger portions of mitochondrial genomes. The remaining genes in the retrieved list and their relative accession frequency distribution (see the complete list in Additional file 1) demonstrate that many regions of the genome were investigated.

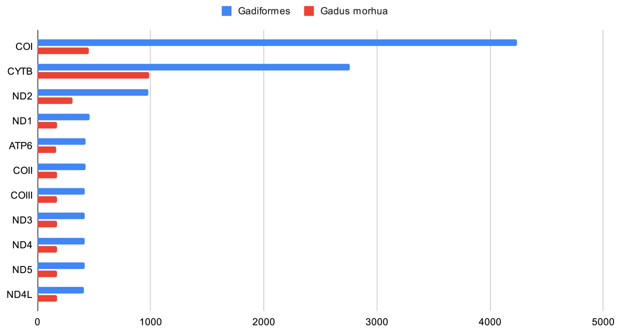

Figure 2. Gene distribution of accessions with available ‘gene’ tag information among *Gadus morhua* and Gadiformes taxons.

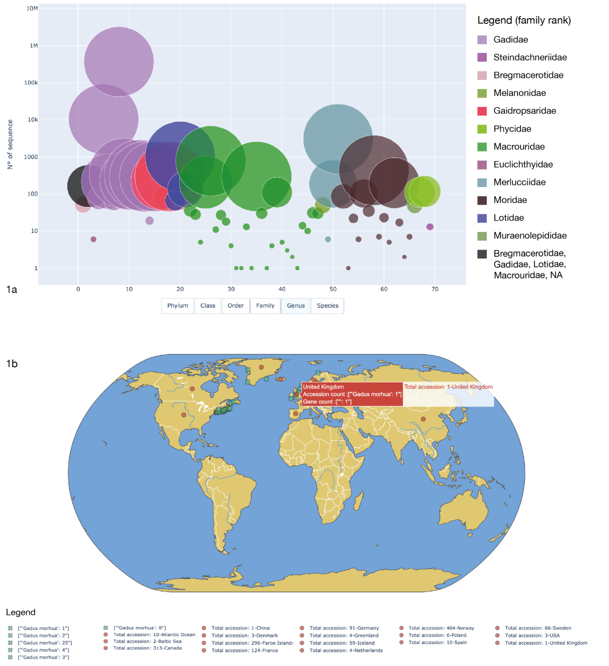

Figure 3. 3a) Scatter plot of Gadiformes accessions representing sequence abundances among families; 3b) World map plot of *Gadus morhua* distribution considering geographic metadata extracted from the records.

Regarding the geographic area, the Gadidae family has a circumpolar distribution, comprising species occurring principally in northern and cool seas [30]. Further, as reported by Jorde and colleagues (2018), in Norway we can recognize four distinct stocks of the Atlantic cod: (1) the oceanic Northeast Arctic cod, (2) coastal cod north of 62°N, (3) coastal cod south of 62°N, and (4) a North Sea/Skagerrak stock, the most densely populated region in Norway [30]. This geographic distribution is partly visible via the metadata extracted by ExTaxsI, as shown in the world map plot in Figure 3b (Additional file 2).

The second scenario takes as an example the Gadiformes Order (phylum: Chordata; class: Actinopterygii), a major group of organisms belonging to marine fisheries. It includes many important food fishes, variously marketed as cods, hakes, grenadiers, moras, moray cods, pelagic cods, codlets and eucla cods [35]. As a vast group, it comprises more than 500 species, which contribute to more than a quarter of the world’s marine fish catch [35, 36].

Via ExTaxsI, this order was explored using the following query “txid8043[ORGN]”, yielding 389,640 accessions (where

8043 is the specific Gadiformes NCBI TaxID; June 21st, 2021), where 61,249 showed the 'gene' tag information. As a group spread on different taxonomic levels, both taxonomy and gene lists were created. In details, in order to explore taxa distribution and accessions abundances across the entire order, the tool created scatter plot and sunburst plot in HTML format. In Figure 3a genera across families is documented in scatter plot modality, while sunburst plot and entirely interactive plots showing the complete dataset are available in the Supplementary Material section (Additional files 3 and 4).

As shown in Figure 3a, Gadidae is the most abundant family represented by 381,460 accessions, followed by Merlucciidae (3,252) and Macrouridae (1,673). These results are in accordance with the literature, as Gadidae family is a primary marine, bottom-dwelling family of fishes in the Gadiformes order with great commercial power [35, 31].

Further, considering the scatter plot in Additional file 3, the interactive visualization allowed to visualize the taxonomy distribution among the accessions available, changing dynamically the rank to explore. This feature permitted to disclose that the genus Gadus is the most abundant of the entire dataset, in which 94.3% of the accessions corresponded to *Gadus morhua* species. This is an expected result, as *Gadus morhua* is documented to be a key species both in the North Atlantic ecosystem and commercial fisheries, with an increasing aquaculture production in several countries [30].

Considering the genetic information reached by ExTaxsI, a total of 28,850 unique genes were found from the 61,249 completely tagged accessions. A representation of the most ten abundant genes is reported in Figure 2, where at the first position COI gene is placed, a widely used marker gene in DNA metabarcoding projects [31], dealing mainly with animal species identification [1], followed by CYTB and ND2 [1].

Finally, these two case studies showed the ability of the tool to accurately portrait the state of the art of the genetic information available in NCBI. Comparing the most abundant genes found among the records, it is possible to see a thin discrepancy between the two taxa explored (Figure 2), highlighting the disclosures that the survey can report. In general, the detection of mitochondrial genes, coding for COI and CYTB, is in accordance with the reliability of these DNA barcodes, principally used in the discrimination of animal species [37, 38, 39]. To date, considering the subjects of our use cases, different studies have used COI or CYTB barcoding to identify seafood products and explore broad patterns in fish mislabelling [40, 41, 42, 43, 44, 45, 46].

In addition, these use cases higlighted the importance of extracting the geographical metadata from NCBI records. The completeness and the collection of such data can improve drastically the biogeographic and ecological research, allowing not only to explore sampling areas, but also to improve phylogeography investigations, biodiversity monitoring and environmental genomics strategies [1, 47]. Moreover, the retrieved data showed an unbalance between the number of records and the number of explorable genes, which is in some cases due to the incompleteness of the 'gene' tag. In the very recent years, genome sequences started to play a key role in public repositories, making sequences available for sharing and reuse. Submission process can be challenging and errors can affect the availability and the quality of the data. For this reason, there is a wide interest to integrate standardized procedures into the annotation process [48] that can be enhanced by adopting FAIR principles and best practices to avoid the error propagation in sequence databases [49, 50], making the data fully explorable in the future.

Explore biodiversity data in pandemic outbreak: the case of SARS-CoV-2

The severe acute respiratory syndrome coronavirus 2 (*SARS-CoV-2*) is an enveloped, positive-sense, single-stranded RNA virus that causes coronavirus disease 2019 (COVID-19). RNA and structural proteins are included into virus particles mediating host cell invasion. After cell infection, RNA encodes structural proteins that make up virus particles. Virus assembly, transcription, replication and host control are mediated by nonstructural proteins [51]. The pandemic linked to SARS-CoV-2 highlighted hidden virus reservoirs in wild animals and their potential to occasionally spillover into human populations [51]. A detailed understanding of this process is crucial to prevent future spillover events. As reported in the seminal paper of Andersen and colleagues (2020) [52], the risk of future reemergence events increases if SARS-CoV-2 pre-adapted in another animal species. SARS-CoV-2 probably originated from *Rhinolophus affinis* bats, with pangolin (*Manis javanica*) as intermediate host [52]. Recently, other animal species were supposed to be possible intermediate hosts between bats and humans (Liu et al., 2020; Zhou and Shi, 2021). To date, ACE2 (Angiotensin-converting enzyme 2), the receptor which binds to the receptor-binding domain (RBD) of SARS-CoV-2 S protein [53], is reported as crucial in host invasion.

To test our approach and explore the genetic information available in NCBI, we decided to extrapolate information of the ACE2 gene from the Vertebrata taxonomic group, with the following query: "txid7742[ORGN] AND ACE2[gene]" (where 7742 is the specific Vertebrata NCBI TaxID). The results show that the ACE2 gene is widely distributed throughout Vertebrata as we obtained a total of 1,391 accessions (June 20, 2021), distributed mainly among the Mammalian Class, with a high representation in Actinopteri and Aves groups (Figure 4a; Additional files 5 and 6 for an interactive exploration). In details, Chiroptera, Primates and Rodentia orders are the most represented, with 126, 125 and 81 accessions respectively. In support of this molecular data survey, Luan and colleague (2020) [54] analyzed the affinity of the 20 key amino acid residues in ACE2 to S protein from mammal, bird, turtle, and snake, and suggested that Bovidae (class: Mammalia) and Cricetidae (order: Rodentia) families should be included in the screening of intermediate hosts for *SARS-CoV-2*. In addition, thanks to the analysis of spike glycoprotein sequences from different animals, the study of Dabravolski and Kavalionak (2020) [55] suggested that the human SARS-CoV-2 could also come from yak (family: Bovidae) as an intermediate host. In this context, ExTaxsI has the advantage to provide the complete list of taxa, allowing an exhaustive exploratory research by downloading all the sequences available for the query input, generating in turn the input for downstream analyses, such as the calculation of sequence similarities among different taxa. Further, investigating shared features with other species can have important implications for understanding potential natural reservoirs, zoonotic transmission, and human-to-animal transmission. Noteworthy, the survey can give researchers an instrument to download specific data related to Covid-19, with a user-friendly approach, to explore interactively the data, including biodiversity related information, and to design informed scientific experiments.

Lastly, we explored the data available for *SARS-CoV-2* (Figure 4) using the following query "txid2697049" (where 2697049 is the specific Severe Acute Respiratory Syndrome Coronavirus 2 NCBI TaxID). Figure 4c shows the top ten most abundant genes found in the retrieved entries and corresponding to a total of 773,293 accessions (June 28, 2021). In particular, the most represented genes are: S (59,506) the spike or surface glycoprotein fragment, ORF1AB (58,872), followed by M (58,867), ORF3A (58,867) and N fragments (58,865) the nu-

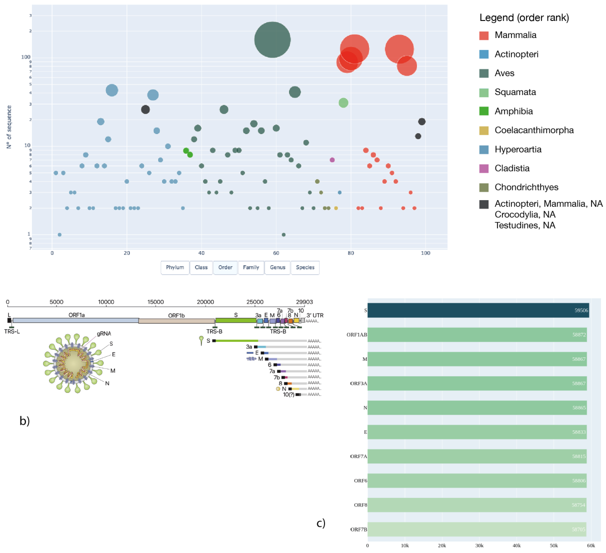

Figure 4. 4a) Scatter plot of ACE2 accessions representing sequence abundances among taxa at order level; 4b) *SARS-CoV-2* representation, from [63]; 4c) gene distribution across accessions of *SARS-CoV-2* data.

cleocapsid protein. These results are in line with the recently published scientific data highlighting the functional aspects of viral proteins. Considering the ORF1AB, several studies demonstrated its pivotal role among coronaviruses [56], providing a clinical target to break down *SARS-CoV-2* infection [57]. In addition, the nucleocapsid phosphoprotein is involved in packaging the RNA into virus particles and protects the viral genome. For these reasons, it has been widely studied and suggested as an antiviral drug target [58, 59]. The spike glycoprotein, instead, is located outside the virus particle, mediating its attachment and promoting the entry into the host cell. It also gives viruses their crown-like appearance. In the very last research, the S protein was found as an important target for diagnostic antigen-based tests, antibody therapies and vaccine development [60, 61]. The entry of *SARS-CoV-2* into host cells is mediated by further processes, for example the activity of the protease TMPRSS2 [62]. Also in this case, the use of ExTaxsI can unearth similar proteases in possible intermediate hosts, revealing new insights into the mechanism of infection.

As also documented in Khailany et al., 2020 [57], the emergent and huge amounts of data collected in the last few months necessitates a large scale exploration. The rapid increment of data releases may give some important insights about *SARS-CoV-2* behaviour in its host species, helping to improve not only our knowledge, but also the design of appropriate prediction models of COVID-19 outbreaks and new target drugs.

Conclusions and future directions

ExTaxsI provides an easy-to-use standalone tool able to interact with NCBI databases and personal datasets, offering instruments to standardize taxonomy information and visualize vast amount of data distributed on different taxonomic levels. It also provides interactive visualization plots, easily shareable through HTML formats.

The user-oriented interrogation of NCBI databases may help researchers involved in environmental genomics fields, from phylogeographic studies to DNA metabarcoding surveys, and also in projects related to human health, as demonstrated with the *SARS-CoV-2* case study.

With this work, we hope to meet the needs of a broad group of researchers, providing an instrument easy to install either on common laptops or on high performance servers and directly connected with NCBI databases. In parallel to the command-line tool, a python library containing all ExTaxsI functions has been implemented, favoring a direct incorporation of such functions into data analysis and exploration pipelines.

In addition, as data volume is increasing over time and NCBI databases still have a few constraints regarding the queries results dimension and their retrieval time required, an automatic management of large queries will be organized in future releases. Finally, we will also consider further data visualization strategies and additional metadata (e.g. GBIF country information) to enhance data interpretation and to provide comprehensive sets of relevant scientific-focused information. In our opinion, ExTaxsI data management ability with its visual interactive exploration can really improve the experimental design phase and the awareness of the information available, facilitating data examination and sharing.

Implementation

ExTaxsI is a bioinformatic tool aimed to explore, elaborate and visualize molecular and taxonomic information via a simple user interface without specific bioinformatic or programming skills. The tool can be run, via command line interface, where the user is guided by the appropriate documentation of each script, avoiding the implementation of ad hoc python code. ExTaxsI is developed in three separate modules, which can be used either interconnected as workflow or independent according to the user needs. The main modules are listed as follows: i) Database creation, ii) Visualization and iii) Taxonomy ID converter.

ExTaxsI is also available as a python library that can be installed through `pip` (package installer for Python), containing the same functions and parameters as those of the command-line tool. A detailed description of each module is provided below.

1 - Database creation module

The module 'Database' allows the user to create multi FASTA files composed of nucleotide sequences, taxonomic lists, genes names and their related accessions, starting from either a single or a batch query mode using csv/tsv input files (Figure 1). After indicating the input type, it is possible to integrate the query with one or more gene name/s (or other details). This step allows to restrict the search in NCBI databases if needed. In general, the output formats are i) a multi-FASTA file (widely used format for molecular sequences) and ii) text file in TSV format, with two columns composed by the accessions code followed by the taxonomy path of each accession at the six main levels separated by semicolons: phylum, class, order, family, genus and species. When requested by the user, the output file of genes names is provided in TSV format consisting of a table with two columns, the first is the list of genes and the other is the frequency values of the respective genes found in the retrieved records. The tool also provides a summary table containing the most popular genes from a list of NCBI taxids, accessions or organisms. In addition, it is possible to create a barplot with the top ten of the summary table, downloadable as a PNG file.

2 - Visualization module

The module 'Visualization' allows the user to create interactive plots, starting from the 'Database' module output or from external sources such as local files (e.g., Additional files 3, 4 and 5) containing taxonomic lists. Before producing the plots, a dialogue box will ask the user to choose a filter value on the data based on the frequency. If the chosen filter value is 0, the tool processes all the data. Otherwise, all the taxonomic units that have not reached the minimum value are inserted into an additional text file, specifically created with a name containing the filter used.

The available plots generated by ExTaxsI are i) scatter plot (Additional file 3), ii) sunburst plot (Additional file 4) and iii) world map plot (Additional file 2). All figures created by the Visualization module can be downloaded as HTML format files. In details, scatter plot uses taxonomy as input to produce a graph that indicates the quantity of each individual taxonomic unit; the interactive plot enables the user to i) choose the taxonomic level to be displayed using the buttons located under the graph and ii) hover over points to show details, such as the number of records within taxa, names of selected taxa and name of the parent taxon. The plot allows also to compare more data on mouse-over, highlight an area of interest with zoom function and view of a specific group or remove specific taxa from the graph. Sunburst plot, instead, from a taxonomy input creates an expansion pie that allows exploring taxonomy by clicking on the taxonomic group of interest and showing the underlying taxa within a new sunburst plot. Also in this case, hovering over points shows the number of records within taxa. Regarding world map plot, the initial input is processed in order to obtain geographic data. The tool exploits the 'Country' metadata stored in the NCBI records to produce a map indicating the position of each entry. In this step, based on the type of geographic data obtained, ExTaxsI divides results into two different arrays: i) a specific array of coordinates (if the coordinates are present in the record) or ii) a specific array of country names (if the coordinates are absent). It is also possible to add external sources data to the map. In each created map, the coordinates are indicated by green X signs, while countries by red circles. Thinking of multiple taxa plotting, each symbol can have a legend that summarizes the data downloaded with the same country name or coordinates description. Further, it is possible to see both genes and counts available among the represented accessions.

3 - Taxonomy ID converter module

This module allows to convert NCBI TaxID into the main six taxonomy ranks and vice versa (phylum, class, order, family, genus and species); it can convert single manual inputs or multiple inputs from a tsv/csv file containing a list of TaxIDs.

Availability of source code and requirements

Command-line tool

No specific system requirements are needed for the installation of ExTaxsI, however for the correct functioning of the software we suggest a minimum of 4GB of RAM. To successfully run ExTaxsI, the following python libraries must be installed: Biopython [28], NumPy [64]), SciPy [65], Matplotlib [66], ipython [67], Pandas [68], SymPy (https://www.sympy.org/en/index.html), nose (https://nose.readthedocs.io/en/latest/), genutils (https://pypi.org/project/genutils/), requests [69] and Plotly (https://plotly.com/), in addition to Plotly-Orca and ETE toolkit [20]. To install all the dependencies compatible versions, we provide a requirement list at the GitHub page https://github.com/qLSLab/ExTaxsI, with a detailed guideline to set directly a conda environment.

Python library

The Python library `extaxsi` is available both in the Github page: https://github.com/qLSLab/ExTaxsI/tree/master/library and in PyPI repository: https://pypi.org/project/extaxsi/

- Project name: ExTaxsI
- Project home page: https://github.com/qLSLab/extaxsi; https://github.com/qLSLab/ExTaxsI/tree/master/library; https://pypi.org/project/extaxsi/
- Operating system(s): Platform independent
- Programming language: Python
- License: GNU GPL version 3

Availability of supporting data and materials

Additional Files

Additional file 1: Gene list in TSV format obtained through ExTaxsI for the species *Gadus morhua*. Gene counts were extracted from 367,455 accessions (query: "txid8049[ORGN]"; 18 of June, 2021).
Additional file 2: World map plot in HTML format created via ExTaxsI extracting the values of 'Country' tag contained in 367,4553 accessions of *Gadus morhua* (query: "txid8049[ORGN]"; 18 of June, 2021). Coordinates are indicated by green X signs, while States by red circles.
Additional file 3: Scatterplot in HTML format created via ExTaxsI extracting the taxonomy of 389,640 accessions of Gadiformes Order (txid8043[ORGN]"; 21 of June, 2021).
Additional file 4: Sunburst plot in HTML format created via ExTaxsI extracting the taxonomy of 388,603 accessions of Gadiformes order (txid8043[ORGN]"; 21 of June, 2021).
Additional file 5: Scatterplot in HTML format created via ExTaxsI extracting the taxonomy related to 1,391 accessions of ACE2 genes belonging to the Vertebrata taxonomic group (query: "txid7742[ORGN] AND ACE2[gene]"; 20 of June, 2021).
Additional file 6: Sunburst plot in HTML format created via ExTaxsI extracting the taxonomy related to 1,391 accessions of ACE2 genes belonging to the Vertebrata taxonomic group (query: "txid7742[ORGN] AND ACE2[gene]"; 20 of June, 2021).

Declarations

List of abbreviations

SILVA: High quality ribosomal RNA databases; BOLD: Barcode of Life Data System; UNITE: Database and sequence management environment centered on the eukaryotic nuclear ribosomal ITS region; ETE: Environment for Tree Exploration; QIIME2: Quantitative Insights Into Microbial Ecology; FASTA: Text-based format for representing either nucleotide sequences or peptide sequences; TAXID: Taxonomy ID; HTML: Hyper-Text Markup Language; COI: Cytochrome Oxidase I; COI: Cytochrome Oxidase II; COIII: Cytochrome Oxidase III; CYTB: Cytochrome B; ND2: NADH dehydrogenase 2; ACE2: Angiotensin-Converting enzyme 2; RBD: Receptor-Binding Domain; PNG: Portable Network Graphics; NCBI: National Center for Biotechnology Information; ENA: European Nucleotide Archive

Consent for publication

Not applicable.

Competing Interests

The authors declare that they have no competing interests.

Funding

This study was funded by the 'Ministero dell'Istruzione dell'Università e della Ricerca' (MIUR) within the project: 'Sistemi Alimentari e Sviluppo Sostenibile—tra ricerca e processi internazionali e africani'. CUP: H42F16002450001. The funder had no role in conducting the research and/or during the preparation of the article.

Author's Contributions

Giulia Agostinetto: Conceptualization, Investigation, Software development, Visualization, Original Draft Preparation, Review, Editing, Supervision, Project Administration. **Alberto Brusati**: Investigation, Software development, Visualization, Review & Editing. **Anna Sandionigi**: Conceptualization, Original Draft Preparation, Review & Editing, Supervision, Project Administration. **Adam Chahed**: Software development, Visualization. **Elena Parladori**: Software development, Visualization. **Bachir Balech**: Review & Editing, Validation. **Antonia Bruno**: Review & Editing, Validation. **Dario Pescini**: Review & Editing, Supervision. **Maurizio Casiraghi**: Funding Acquisition, Supervision. All authors read and approved the final manuscript, contributing critically important comments.

Acknowledgements

The authors thank all the ELIXIR Biodiversity community members for the support and all researchers who have provided input on the development of ExTaxsI project.

References

1. Porter TM, Hajibabaei M. Scaling up: A guide to high-throughput genomic approaches for biodiversity analysis. Molecular ecology 2018;27(2):313–338.
2. Ruppert KM, Kline RJ, Rahman MS. Past, present, and future perspectives of environmental DNA (eDNA) metabarcoding: A systematic review in methods, monitoring, and applications of global eDNA. Global Ecology and Conservation 2019;17:e00547.
3. Deiner K, Bik HM, Mächler E, Seymour M, Lacoursière-Roussel A, Altermatt F, et al. Environmental DNA metabarcoding: Transforming how we survey animal and plant communities. Molecular ecology 2017;26(21):5872–5895.
4. Hampton SE, Jones MB, Wasser LA, Schildhauer MP, Supp SR, Brun J, et al. Skills and knowledge for data-intensive environmental research. BioScience 2017;67(6):546–557.
5. White EP, Baldridge E, Brym ZT, Locey KJ, McGlinn DJ, Supp SR. Nine simple ways to make it easier to (re) use your data. Ideas in Ecology and Evolution 2013;6(2).
6. Michener WK, Jones MB. Ecoinformatics: supporting ecology as a data-intensive science. Trends in ecology & evolution 2012;27(2):85–93.
7. Mitchell AL, Almeida A, Beracochea M, Boland M, Burgin J, Cochrane G, et al. MGnify: the microbiome analysis resource in 2020. Nucleic acids research 2020;48(D1):D570–D578.
8. Almeida A, Mitchell AL, Boland M, Forster SC, Gloor GB, Tarkowska A, et al. A new genomic blueprint of the human gut microbiota. Nature 2019;568(7753):499–504.
9. Kaur P, Klan F, König-Ries B. Issues and Suggestions for the Development of a Biodiversity Data Visualization Support Tool. In: EuroVis (Short Papers); 2018. p. 73–77.
10. Hardisty A, Roberts D, et al. A decadal view of biodiversity informatics: challenges and priorities. BMC ecology 2013;13(1):16.
11. Pruesse E, Quast C, Knittel K, Fuchs BM, Ludwig W, Peplies J, et al. SILVA: a comprehensive online resource for quality checked and aligned ribosomal RNA sequence data compatible with ARB. Nucleic acids research 2007;35(21):7188–7196.
12. Ratnasingham S, Hebert PD. BOLD: The Barcode of Life Data System (http://www. barcodinglife. org). Molecular ecology notes 2007;7(3):355–364.
13. Nilsson RH, Larsson KH, Taylor AFS, Bengtsson-Palme J, Jeppesen TS, Schigel D, et al. The UNITE database for molecular identification of fungi: handling dark taxa and parallel taxonomic classifications. Nucleic acids research 2019;47(D1):D259–D264.
14. Keller A, Hohlfeld S, Kolter A, Schultz J, Gemeinholzer B, Ankenbrand MJ. BCdatabaser: on-the-fly reference database creation for (meta-) barcoding. Bioinformatics 2020;36(8):2630–2631.
15. Ankenbrand MJ, Keller A, Wolf M, Schultz J, Förster F. ITS2 database V: Twice as much. Molecular Biology and Evolution 2015;32(11):3030–3032.
16. Benson D, Karsch-Mizrachi I, Lipman D, Ostell J, Wheeler D. GenBank Nucleic Acids Res. jan 2008;1:33.
17. Eaton K. NCBImeta: Efficient and comprehensive metadata retrieval from NCBI databases. Journal of Open Source Software 2020;5(46):1990.
18. Federhen S. The NCBI taxonomy database. Nucleic acids research 2012;40(D1):D136–D143.
19. Macher TH, Beermann AJ, Leese F. TaxonTableTools: A comprehensive, platform-independent graphical user interface software to explore and visualise DNA metabarcoding data. Molecular Ecology Resources 2021;.
20. Huerta-Cepas J, Serra F, Bork P. ETE 3: reconstruction, analysis, and visualization of phylogenomic data. Molecular biology and evolution 2016;33(6):1635–1638.
21. Bolyen E, Rideout JR, Dillon MR, Bokulich NA, Abnet CC, Al-Ghalith GA, et al. Reproducible, interactive, scalable and extensible microbiome data science using QIIME 2. Nature biotechnology 2019;37(8):852–857.
22. Rognes T, Flouri T, Nichols B, Quince C, Mahé F. VSEARCH: a versatile open source tool for metagenomics. PeerJ 2016;4:e2584.
23. Bengtsson-Palme J, Hartmann M, Eriksson KM, Pal C, Thorell K, Larsson DGJ, et al. METAXA2: improved identification and taxonomic classification of small and large subunit rRNA in metagenomic data. Molecular ecology resources 2015;15(6):1403–1414.
24. Mahé F, Rognes T, Quince C, de Vargas C, Dunthorn M. Swarm v2: highly-scalable and high-resolution amplicon clustering. PeerJ 2015;3:e1420.
25. Camacho C, Coulouris G, Avagyan V, Ma N, Papadopoulos J, Bealer K, et al. BLAST+: architecture and applications. BMC bioinformatics 2009;10(1):421.
26. Wang Q, Garrity GM, Tiedje JM, Cole JR. Naive Bayesian classifier for rapid assignment of rRNA sequences into the new bacterial taxonomy. Applied and environmental microbiology 2007;73(16):5261–5267.
27. Coordinators NR. Database resources of the national cen-

ter for biotechnology information. Nucleic acids research 2014;42(D1):D7–D17.
28. Cock PJ, Antao T, Chang JT, Chapman BA, Cox CJ, Dalke A, et al. Biopython: freely available Python tools for computational molecular biology and bioinformatics. Bioinformatics 2009;25(11):1422–1423.
29. Blomberg N, Lauer KB. Connecting data, tools and people across Europe: ELIXIR's response to the COVID-19 pandemic. European Journal of Human Genetics 2020;p. 1–5.
30. Jorde PE, Kleiven AR, Sodeland M, Olsen EM, Ferter K, Jentoft S, et al. Who is fishing on what stock: population-of-origin of individual cod (Gadus morhua) in commercial and recreational fisheries. ICES Journal of Marine Science 2018;75(6):2153–2162.
31. Knudsen SW, Ebert RB, Hesselsøe M, Kuntke F, Hassingboe J, Mortensen PB, et al. Species-specific detection and quantification of environmental DNA from marine fishes in the Baltic Sea. Journal of experimental marine biology and ecology 2019;510:31–45.
32. Star B, Nederbragt AJ, Jentoft S, Grimholt U, Malmstrøm M, Gregers TF, et al. The genome sequence of Atlantic cod reveals a unique immune system. Nature 2011;477(7363):207–210.
33. Kurlansky M, Davidson RM. Cod: a Biography of the Fish that Changed the world. Phoenix Books; 2006.
34. Johansen SD, Coucheron DH, Andreassen M, Karlsen BO, Furmanek T, Jørgensen TE, et al. Large-scale sequence analyses of Atlantic cod. New Biotechnology 2009;25(5):263–271.
35. Nelson JS, Grande TC, Wilson MV. Fishes of the World. John Wiley & Sons; 2016.
36. Costello MJ, Bouchet P, Boxshall G, Fauchald K, Gordon D, Hoeksema BW, et al. Global coordination and standardisation in marine biodiversity through the World Register of Marine Species (WoRMS) and related databases. PloS one 2013;8(1):e51629.
37. Hebert PD, Ratnasingham S, De Waard JR. Barcoding animal life: cytochrome c oxidase subunit 1 divergences among closely related species. Proceedings of the Royal Society of London Series B: Biological Sciences 2003;270(suppl_1):S96–S99.
38. Hellberg RS, Kawalek MD, Van KT, Shen Y, Williams-Hill DM. Comparison of DNA extraction and PCR setup methods for use in high-throughput DNA barcoding of fish species. Food analytical methods 2014;7(10):1950–1959.
39. Mueller S, Handy SM, Deeds JR, George GO, Broadhead WJ, Pugh SE, et al. Development of a COX1 based PCR-RFLP method for fish species identification. Food Control 2015;55:39–42.
40. Fernandes TJ, Costa J, Oliveira MBP, Mafra I. DNA barcoding coupled to HRM analysis as a new and simple tool for the authentication of Gadidae fish species. Food Chemistry 2017;230:49–57.
41. Cline E. Marketplace substitution of Atlantic salmon for Pacific salmon in Washington State detected by DNA barcoding. Food Research International 2012;45(1):388–393.
42. Di Pinto A, Di Pinto P, Terio V, Bozzo G, Bonerba E, Ceci E, et al. DNA barcoding for detecting market substitution in salted cod fillets and battered cod chunks. Food chemistry 2013;141(3):1757–1762.
43. Miller DD, Mariani S. Smoke, mirrors, and mislabeled cod: poor transparency in the European seafood industry. Frontiers in Ecology and the Environment 2010;8(10):517–521.
44. Rasmussen RS, Morrissey MT. DNA-based methods for the identification of commercial fish and seafood species. Comprehensive reviews in food science and food safety 2008;7(3):280–295.
45. Wong EHK, Hanner RH. DNA barcoding detects market substitution in North American seafood. Food Research International 2008;41(8):828–837.
46. Yancy HF, Zemlak TS, Mason JA, Washington JD, Tenge BJ, Nguyen NLT, et al. Potential use of DNA barcodes in regulatory science: applications of the Regulatory Fish Encyclopedia. Journal of Food Protection 2008;71(1):210–217.
47. Cordier T, Alonso-Sáez L, Apothéloz-Perret-Gentil L, Aylagas E, Bohan DA, Bouchez A, et al. Ecosystems monitoring powered by environmental genomics: a review of current strategies with an implementation roadmap. Molecular Ecology 2020;.
48. Geib SM, Hall B, Derego T, Bremer FT, Cannoles K, Sim SB. Genome Annotation Generator: a simple tool for generating and correcting WGS annotation tables for NCBI submission. GigaScience 2018;7(4):giy018.
49. Wilkinson MD, Dumontier M, Aalbersberg IJ, Appleton G, Axton M, Baak A, et al. The FAIR Guiding Principles for scientific data management and stewardship. Scientific data 2016;3(1):1–9.
50. Pirovano W, Boetzer M, Derks MF, Smit S. NCBI-compliant genome submissions: tips and tricks to save time and money. Briefings in Bioinformatics 2017;18(2):179–182.
51. Lu R, Zhao X, Li J, Niu P, Yang B, Wu H, et al. Genomic characterisation and epidemiology of 2019 novel coronavirus: implications for virus origins and receptor binding. The Lancet 2020;395(10224):565–574.
52. Andersen KG, Rambaut A, Lipkin WI, Holmes EC, Garry RF. The proximal origin of SARS-CoV-2. Nature medicine 2020;26(4):450–452.
53. Letko M, Marzi A, Munster V. Functional assessment of cell entry and receptor usage for SARS-CoV-2 and other lineage B betacoronaviruses. Nature microbiology 2020;5(4):562–569.
54. Luan J, Jin X, Lu Y, Zhang L. SARS-CoV-2 spike protein favors ACE2 from Bovidae and Cricetidae. Journal of medical virology 2020;.
55. Dabravolski SA, Kavalionak YK. SARS-CoV-2: Structural diversity, phylogeny, and potential animal host identification of spike glycoprotein. Journal of medical virology 2020;.
56. Wan Y, Shang J, Graham R, Baric RS, Li F. Receptor recognition by the novel coronavirus from Wuhan: an analysis based on decade-long structural studies of SARS coronavirus. Journal of virology 2020;94(7).
57. Khailany RA, Safdar M, Ozaslan M. Genomic characterization of a novel SARS-CoV-2. Gene reports 2020;p. 100682.
58. Wu F, Zhao S, Yu B, Chen YM, Wang W, Song ZG, et al. A new coronavirus associated with human respiratory disease in China. Nature 2020;579(7798):265–269.
59. Gordon DE, Jang GM, Bouhaddou M, Xu J, Obernier K, White KM, et al. A SARS-CoV-2 protein interaction map reveals targets for drug repurposing. Nature 2020;p. 1–13.
60. Salvatori G, Luberto L, Maffei M, Aurisicchio L, Roscilli G, Palombo F, et al. SARS-CoV-2 SPIKE PROTEIN: an optimal immunological target for vaccines. Journal of Translational Medicine 2020;18:1–3.
61. Pillay TS. Gene of the month: the 2019-nCoV/SARS-CoV-2 novel coronavirus spike protein. Journal of Clinical Pathology 2020;.
62. Hoffmann M, Kleine-Weber H, Schroeder S, Krüger N, Herrler T, Erichsen S, et al. SARS-CoV-2 cell entry depends on ACE2 and TMPRSS2 and is blocked by a clinically proven protease inhibitor. Cell 2020;.
63. Kim D, Lee JY, Yang JS, Kim JW, Kim VN, Chang H. The architecture of SARS-CoV-2 transcriptome. Cell 2020;.
64. Harris CR, Millman KJ, van der Walt SJ, Gommers R, Virtanen P, Cournapeau D, et al. Array programming with

NumPy. Nature 2020;585(7825):357–362.
65. Virtanen P, Gommers R, Oliphant TE, Haberland M, Reddy T, Cournapeau D, et al. SciPy 1.0: fundamental algorithms for scientific computing in Python. Nature methods 2020;17(3):261–272.
66. Hunter JD. Matplotlib: A 2D graphics environment. Computing in science & engineering 2007;9(03):90–95.
67. Pérez F, Granger BE. IPython: a system for interactive scientific computing. Computing in science & engineering 2007;9(3):21–29.
68. McKinney W, et al. pandas: a foundational Python library for data analysis and statistics. Python for high performance and scientific computing 2011;14(9):1–9.
69. Chandra RV, Varanasi BS. Python requests essentials. Packt Publishing Ltd; 2015.

Gene list in TSV format obtained throughExTaxsI for the speciesGadus morhua.

Click here to access/download
Supplementary Material
additional_file_1.tsv

World map plot in HTML format created via ExTaxsI extracting the values of ’Country’ tag con-tained into 367,4553 accessions of

Click here to access/download
Supplementary Material
additional_file_2.html

catterplot in HTML format created viaExTaxsI extracting the
taxonomy of 389,640 accessions of Gadiformes Order

Click here to access/download
Supplementary Material
additional_file_3.html

catterplot in HTML format created viaExTaxsI extracting the
taxonomy related to 1,391 accessionsof ACE2 genes belonging to

Click here to access/download
Supplementary Material
additional_file_4.html

catterplot in HTML format created viaExTaxsI extracting the
taxonomy related to 1,391 accessionsof ACE2 genes belonging to

Click here to access/download
Supplementary Material
additional_file_5.html

Sunburst plot in HTML format created viaExTaxsI extracting the
taxonomy related to 1,391 accessionsof ACE2 genes belonging to

Click here to access/download
Supplementary Material
additional_file_6.html

reply to reviewers attached to the cover letter
Click here to access/download;Personal Cover;Answer_revision_submission GIGA-D-20-00337.docx

Reviewer #1
This seems to be an interesting visualization software, but the software required more work. See attached PDF and screenshots for details (PDF copy and paste below).

I tried running the software, and I believe it requires some improvement before being released. There were multiple errors starting with the installation, and, once I overcame those, with running. Generally need better documentation on the GitHub repo, example files, a tutorial-style walkthrough -- a Jupyter notebook would be best.

We thank the reviewer for this comment, during the last months we have rewritten all the documentation present in Github:

https://github.com/qLSLab/ExTaxsI

New example files are added and a tutorial in Markdown is now available in the example folder in Github:

https://github.com/qLSLab/ExTaxsI/blob/master/examples/tutorial.md

Platform: Linux Ubuntu 18.04, conda 4.9.2

Major revisions:
1. Need more detail on the NCBI API key: this is not an obvious thing for most people. Perhaps link to the page at NCBI that explains it.

We thank the reviewer for this comment, it is now possible to find the requested information in the README paragraph:
“Installation instructions in a nutshell” and in the tutorial “Before starting” the detailed instruction for the NCBI API set:

https://github.com/qLSLab/ExTaxsI/blob/master/examples/tutorial.md

2. Need more detail on editing the configuration file. Took me a while to understand how this works. Examples will help.

We thank the reviewer for this suggestion. We implemented in the tutorial section “Before starting” (https://github.com/qLSLab/ExTaxsI/blob/master/examples/tutorial.md) the lines of the configuration file that the user should modify, providing the link to create the NCBI account (please also refer to the previous point for details).

3. Installation is not working as advertised on Githubb site. Missing dependencies are Biopython and ete3.
Thank to review to underline this missing point, we now fixed all the needed dependencies.

4. Taxonomy ID converter seems not to work:

---- TAXONOMY ID CONVERTER MODULE ----
Do you want to convert taxonomy IDs through a file or manual input?
(f > file, m > manual) m
Traceback (most recent call last):
File "ExTaxsI.py", line 3031, in <module>
main_menu()
File "ExTaxsI.py", line 2954, in main_menu
taxonomyID_module()
File "ExTaxsI.py", line 2784, in taxonomyID_module
for counter, taxa in enumerate(taxa_ids):
UnboundLocalError: local variable 'taxa_ids' referenced before
assignment

Thanks to the reviewer to underline this error. Following the applied tests, the function now works correctly.

5. Map creation (first option) gives an error (see below). The second default option seems to create an empty map (see attached file).

Do you want to use a manual search or through a file? (f > file, m >
manual): m
|---
--------|
|--------------------------- *SEARCH FIELD TAGS*
----------------------------|
| *Accession = [ACCN] All Fields = [ALL] Gene Name =*
[GENE] |

| *Organism = [ORGN] Sequence Length = [SLEN] Filter =*
[FILT] |
|---
--------|
Examples of queries:
1) odonata[ORGN] AND (latest[filter] AND all[FILT] NOT
anomalous[filter])
2) txid33208[Organism:exp] AND (18S OR SSU) NOT (mitochondrial OR

complete genome OR whole genome)
No results found with this query: odonata[ORGN] AND (latest[filter] AND all[FILT] NOT anomalous[filter])
Maybe a typo?
Error: Empty ID list; Nothing to store
Retrying in a few seconds

Thanks to the reviewer to underline this point. The function of the first option was correct, we made a mistake suggesting it as an example query that returns an empty list. Now in the tutorial and in the tool guidelines, we added no empty queries as examples.
The second option returned an empty map due to an exception produced by the high number of entries. The function of the map plot was not able to generate a graph with a fitting legend. Now all these limits were removed and the function correctly plots with a split legend fitting correctly the boundaries of the image. In general, we added details regarding the most common 'Warnings' to help users run the tool correctly or adjust the queries.

6. It is unclear what one needs to do here (sunburst plot). Which file format? I could not find an explanation in Github repository. Perhaps supply example files for all these contingencies in the software?

Choose which type of input you will use
Enter one of the following options number
1 - Taxonomy file from this tool
2 - Doing a query on NCBI and directly plot it
3 - Manual created file
>>Enter your choice (only the number): 1
>> Enter the taxonomy file path name :

Thanks to the reviewer to underline this point. We wrote a full explanation of this step in our tutorial, adding an example file format (tsv and csv) specified in the new tutorial that can be downloaded from the "example" folder. In particular, with options 1 and 3 it is possible to use external sources. Option 1 accepts the file with accessions and taxonomy created by the tool, while option 3 accepts as input a tsv or csv file with a 6 rank taxonomy separated by ';' (in detail, see the file 'example_taxonomy_by_user.csv' in 'example' directory).

Reviewer #2
This manuscript proposes a presents a tool called ExTaxsI for management and plotting of molecular and taxonomic data from NCBI. Information can be persisted on a local database as well as FASTA-formatted sequences, which can be used to display the information as scatter or sunburst-pie plots, and maps. The tool uses the Entrez API from NCBI to retrieve data. It also

uses the ETE toolkit to manage taxonomic data. Three use cases were presenting to demonstrate ExTaxsI.

Using ExTaxSi from the command-line apparently produces consistent and correct outputs. Minor errors were found in the installation instructions and are listed at the end of the review. However, ExTaxSi functionality seems to be available only through this command-line interface. This considerably limits the applicability of the tool since many researchers usually incorporate these routines programmatically to their scripts. It would be more useful if ExTaxSi functions were provided additionally through a library that could be imported in Python scripts. This would enable more use cases and would lead to a wider applicability.

We chose to implement only the command line interface because the time computing needed to manage large amounts of data in a graphical version is too high. Our tool does not require any bioinformatics skill and it can be easily used by anyone. The interaction with CLI is guided by our scripts, adding questions to the users that facilitate the usage and does not implicate any python skill (which can be required if a python library is implemented).
After testing the tool with researchers not experts in bioinformatic tools (e.g. microbiologists and zoologists), we thought that a CLI can be the best compromise between function optimization and managing and easy usage. In addition, the Extaxsi tool was tested successfully in all the three operating systems: Windows, macOS and Linux.

The manuscript also lacks a detailed comparison with related implementations, e.g. NCBImeta (DOI: 10.21105/joss.01990), to demonstrate its innovation and unique contribution. For this purpose, it would be important to compare ExTaxsI to similar or related tools used by the community.

We thank the reviewer for this comment. We added in the manuscript (Introduction section) a comparison with similar contributions (e.g. NCBImeta and TaxonTablesTools), highlighting the advantages of our work and the differences between ExTaxsI and the previously published tools.

The README provided on Github has some easy to spot errors:

conda create - -name myenv - -file requirements.txt

Should be

conda create --name myenv --file requirement.txt

We thank the reviewer for this comment, we fixed the text in the README in accordance with the reviewer's suggestions.

pip install -r requirements.txt

Should be

pip install -r requirement.txt

We thank the reviewer for this comment, we corrected the text in the README following the reviewer's suggestions.

Even then, it returns an error:
ERROR: Invalid requirement: 'numpy=1.16.3' (from line 1 of requirement.txt)
Hint: = is not a valid operator. Did you mean == ?

We thank the reviewer for this comment, we have arranged the text in the dependencies file following the reviewer's suggestions.

Also, one needs to install the Biopython library, otherwise the tool cannot find some functions used in the implementation:

```
Traceback (most recent call last):
  File "ExTaxsI.py", line 50, in <module>
    from Bio import Entrez
ModuleNotFoundError: No module named 'Bio'
```

We thank the reviewer for this comment, we expanded the installation section in the tutorial file to better describe the dependencies of the tools.

https://github.com/qLSLab/ExTaxsI/blob/master/examples/tutorial.md
