# Supplementary material for: ExTaxsI: an exploration tool of biodiversity molecular data
Source: Gigascience. 2022 Jan 25;11:giab092. doi: 10.1093/gigascience/giab092 (PMC8848311; doi:10.1093/gigascience/giab092)
Supplement: giab092_Response_to_Reviewer_Comments_Revision_1 [file giab092_response_to_reviewer_comments_revision_1.pdf]

Author's Response To Reviewer Comments

Close

Dear Nicole Nogoy,

Here are the answers to the comments received in the last revision, in the attachment you will find the pdf with underlined the differences between the previous version (30-11, red) and the new one (3-12, green.)

Editor:
Your manuscript "ExTaxsI: an exploration tool of biodiversity molecular data" (GIGA-D-21-00226R1) has been assessed by our reviewers. Based on these reports, and my own assessment as Editor, I am pleased to inform you that it is potentially acceptable for publication in GigaScience, once you have carried out some essential revisions suggested by our Executive Editor.

Authors:
Thank you for following us in the process of reviewing our work, and we are honored to be able to publish on Gigascience

Editor:
Please cite the GigaDB DOI under "Availability of supporting data" and add the DOI citation in the references (new #70). Please see the attached PDF with my marked up comments as to where to add this information.

Authors:
Done and we add the citation in the “Reference” section.

Editor:
In addition, please register any new software application in the bio.tools and SciCrunch.org databases to receive RRID (Research Resource Identification Initiative ID) and biotoolsID identifiers, and include these in your manuscript. This will facilitate tracking, reproducibility and re-use of your tool.

Authors:
Done and we add the ID (RRID) SCR_021846 in the main text in the “Availability of source code and requirement”, subsection “Python library”.

Close
