# Supplementary material for: ExTaxsI: an exploration tool of biodiversity molecular data
Source: Gigascience. 2022 Jan 25;11:giab092. doi: 10.1093/gigascience/giab092 (PMC8848311; doi:10.1093/gigascience/giab092)
Supplement: giab092_Reviewer_2_Report_Original_Submission — Luiz Gadelha -- 9/23/2021 Reviewed [file giab092_reviewer_2_report_original_submission.pdf]

Reviewer Report

Title: ExTaxsI: an exploration tool of biodiversity molecular data

Version: Original Submission **Date:** 9/23/2021

Reviewer name: Luiz Gadelha

Reviewer Comments to Author:

This manuscript proposes a presents a tool called ExTaxsI for management and plotting of molecular and taxonomic data from NCBI. Information can be persisted on a local database as well as FASTA-formatted sequences, which can be used to display the information as scatter or sunburst-pie plots, and maps. The tool uses the Entrez API from NCBI to retrieve data. It also uses the ETE toolkit to manage taxonomic data. Three use cases were presenting to demonstrate ExTaxsI:
- geospatial distribution and gene data of Atlantic cod and the Gadiformes Order,
- exploration of biodiversity data related to the SARS-COV-2 pandemic.

Using ExTaxSi from the command-line apparently produces consistent and correct outputs.
However, ExTaxSi functionality seems to be available only through this command-line interface. This considerably limits the applicability of the tool since many researchers usually incorporate these routines programmatically to their scripts. It would be more useful if ExTaxSi functions were provided additionally through a library that could be imported in Python scripts. This would enable more use cases and would lead to a wider applicability.
Some issues in a previous submission of this manuscript were corrected. A more detailed comparison with related tools is included and the installation instructions for the tool now work correctly. The documentation was also significantly improved.

Level of Interest

Please indicate how interesting you found the manuscript: Choose an item.

Quality of Written English

Please indicate the quality of language in the manuscript: Choose an item.

Declaration of Competing Interests

Please complete a declaration of competing interests, considering the following questions:

- Have you in the past five years received reimbursements, fees, funding, or salary from an organisation that may in any way gain or lose financially from the publication of this manuscript, either now or in the future?

- Do you hold any stocks or shares in an organisation that may in any way gain or lose financially from the publication of this manuscript, either now or in the future?
- Do you hold or are you currently applying for any patents relating to the content of the manuscript?
- Have you received reimbursements, fees, funding, or salary from an organization that holds or has applied for patents relating to the content of the manuscript?
- Do you have any other financial competing interests?
- Do you have any non-financial competing interests in relation to this paper?

If you can answer no to all of the above, write 'I declare that I have no competing interests' below. If your reply is yes to any, please give details below.

I declare that I have no competing interests

I agree to the open peer review policy of the journal. I understand that my name will be included on my report to the authors and, if the manuscript is accepted for publication, my named report including any attachments I upload will be posted on the website along with the authors' responses. I agree for my report to be made available under an Open Access Creative Commons CC-BY license (http://creativecommons.org/licenses/by/4.0/). I understand that any comments which I do not wish to be included in my named report can be included as confidential comments to the editors, which will not be published.

Choose an item.

To further support our reviewers, we have joined with Publons, where you can gain additional credit to further highlight your hard work (see: https://publons.com/journal/530/gigascience). On publication of this paper, your review will be automatically added to Publons, you can then choose whether or not to claim your Publons credit. I understand this statement.

Yes Choose an item.
